# Prediction of Autism at 3 Years from Behavioural and Developmental Measures in High-Risk Infants: A Longitudinal Cross-Domain Classifier Analysis

**DOI:** 10.1007/s10803-018-3509-x

**Published:** 2018-02-16

**Authors:** G. Bussu, E. J. H. Jones, T. Charman, M. H. Johnson, J. K. Buitelaar, the BASIS Team, S. Baron-Cohen, R. Bedford, P. Bolton, A. Blasi, S. Chandler, T. Charman, C. Cheung, K. Davies, M. Elsabbagh, J. Fernandes, I. Gammer, H. Garwood, T. Gliga, J. Guiraud, K. Hudry, M. H. Johnson, M. Liew, S. Lloyd-Fox, H. Maris, L. O’Hara, G. Pasco, A. Pickles, H. Ribeiro, E. Salomone, L. Tucker, A. Volein

**Affiliations:** 1 Donders Institute for Brain, Cognition and Behaviour, Radboud University Medical Centre, Kapittelweg 29, 6525 EN Nijmegen, The Netherlands; 2 Centre for Brain and Cognitive Development, Birkbeck, University of London, 32 Torrington Square, London, WC1E 7JL UK; 3 Department of Psychology, Institute of Psychiatry, Psychology and Neuroscience, King’s College London, De Crespigny Park, Denmark Hill, London, SE5 8AF UK

**Keywords:** Autism, Early prediction, Machine learning, Data integration, Individual prediction, High-risk, Longitudinal study

## Abstract

We integrated multiple behavioural and developmental measures from multiple time-points using machine learning to improve early prediction of individual Autism Spectrum Disorder (ASD) outcome. We examined Mullen Scales of Early Learning, Vineland Adaptive Behavior Scales, and early ASD symptoms between 8 and 36 months in high-risk siblings (HR; n = 161) and low-risk controls (LR; n = 71). Longitudinally, LR and *HR-Typical* showed higher developmental level and functioning, and fewer ASD symptoms than *HR-Atypical* and *HR-ASD*. At 8 months, machine learning classified *HR-ASD* at chance level, and broader atypical development with 69.2% Area Under the Curve (AUC). At 14 months, ASD and broader atypical development were classified with approximately 71% AUC. Thus, prediction of ASD was only possible with moderate accuracy at 14 months.

**Electronic supplementary material:**

The online version of this article (10.1007/s10803-018-3509-x) contains supplementary material, which is available to authorized users.

## Introduction

Although symptoms of Autism Spectrum Disorders (ASD) typically emerge early in life, a reliable diagnosis is usually not achieved before age 3 or later (Steiner et al. 2012). Evidence suggests that the best prognosis for ASD currently lies in early targeted intervention aimed to improve later outcome by modifying emergent atypical developmental trajectories (Fernell et al. 2013; MacDonald et al. 2014). A recent follow-up study on the effects of parent-mediated social communication intervention in infants at high familial risk of ASD between 9 and 14 months shows a treatment effect on symptom severity extended 24 months after intervention end (Green et al. 2017). However, the sustained delivery of behavioural intervention to all infants at risk for ASD based only on traits would be too expensive, and the risk/benefit ratio may be less favourable for infants who would have developed typically anyway. Thus, individual prediction of later development of ASD as soon as early signs emerge could help to better target early intervention strategies.

Limits to detection of ASD before 24 months come from the high heterogeneity of the disorder and the relatively late emergence of the core characteristics of ASD. Heterogeneity in onset, aetiology, phenotype, neurobiology, and developmental trajectory points to multiple underlying processes acting together and leading to the disorder rather than a unitary biological process (Jones et al. 2014; Lai et al. 2013; Vorstman et al. 2017). Therefore, the investigation of different types of data is essential to capture the different aspects of the disorder. Machine learning holds the potential to provide a robust algorithm for prediction of later clinical outcome combining complementary information from different sources in an efficient way, and allowing the identification of the most predictive combination of measures. The application of these methods has already shown promise for classification of children with ASD (Ingalhalikar et al. 2014; Uddin et al. 2013; Wee et al. 2014). In the present study we apply machine learning algorithms to predict clinical outcome at 36 months from different combinations of behavioural and developmental measures at 8 and 14 months. Despite a general consensus on the added value of data integration for prediction of ASD, the method has not previously been applied to behavioural measures and standard developmental assessments.

Research in the early recognition and diagnosis of ASD has been focused on prospective longitudinal studies of infants at high-risk for autism because they have an older sibling with ASD. High-risk infants (HR) have about a 20% risk of developing ASD, significantly higher than the population prevalence of 1.5% (Christensen 2016; Ozonoff et al. 2011; Sandin et al. 2014; Szatmari et al. 2016), and thus the high-risk design allows us to study the early manifestations of the condition and understand the behavioural, cognitive and neural mechanisms that precede the clinical onset of ASD (Jones et al. 2014). Yet, these studies have mainly focused on average differences between infants who later develop ASD and their typically developing peers, measuring group differences by means of p-values. Convergent evidence supports the emergence of overt behavioural markers for ASD by the end of the second year of life, such as atypical eye contact, visual tracking, disengagement of visual attention and orienting to name (Elsabbagh and Johnson 2010; Gliga et al. 2014; Jones et al. 2014; Ozonoff et al. 2010; Rogers 2009; Yirmiya and Charman 2010; Zwaigenbaum et al. 2015). Objectively measured behavioural signs for ASD emerging before 12 months include a fall in fixation to the eye region between 2 and 6 months (Jones and Klin 2013), reduced gaze fixation to people at 6 months (Chawarska et al. 2013), and vocal atypicalities (Paul et al. 2011). But early markers for ASD are not limited to social domains. By 14 months, high-risk siblings developing ASD performed significantly worse than unaffected siblings on all scales of the Mullen Scales of Early Learning (MSEL) except for Visual Reception (Landa and Garrett-Mayer 2006), and impairments in verbal skills (particularly receptive language) (Barbaro and Dissanayake 2012), and motor skills were associated with later diagnosis of ASD (Chawarska et al. 2007; Landa and Garrett-Mayer 2006; Landa et al. 2013; MacDonald et al. 2013), even already by the age of 7 months (Leonard et al., 2014; Libertus et al. 2014). Few studies so far have conducted analyses that combined measures from different domains. Estes and colleagues (2015) investigated trajectories of developmental abilities, as measured by the MSEL; adaptive functioning, as measured by the Vineland Adaptive Behavior Scales (VABS); and early ASD symptoms, as measured by the Autism Observational Scale for Infants (AOSI), in infants at high and low risk for autism in relation to ASD at 24 months, showing a pattern of symptoms starting in the sensorimotor domain at 6 months and moving to the social-communication domain after 12 months. Thus, prediction of autism may require a multi-measure approach.

Although previous findings on group differences between infants who later develop ASD and their typically developing peers are valuable in terms of finding relevant biomarkers for the disorder, there is often substantial overlap between groups in individual variation, making prediction for individual infants difficult. The aim of individual prediction of outcome is to automatically classify each individual into one group (e.g. ASD vs. non-ASD outcome), and performance is usually measured by accuracy or Area Under the Curve (AUC). The AUC is a measure of predictive accuracy computed as the area under the Receiver Operating Characteristic (ROC) curve, which is a plot of true positive rate vs. false positive rate for the model under evaluation (Metz 1978). Prediction at chance level results in 50% AUC, while prediction has moderate accuracy for AUC above 70%. Few studies have used behavioural measures to predict individual outcome of ASD, individual prediction being more focused on neuroimaging data (Arbabshirani et al. 2017; Emerson et al. 2017; Libero et al. 2015; Zhou et al. 2014). Macari and colleagues (2012) employed a decision-tree nonparametric learning algorithm to classify typical versus ‘atypical’ high-risk infants using as features measures from the Autism Diagnostic Observation Schedule (ADOS) at 12 months. ‘Atypicality’ included but was not limited to ASD, and was based on clinical evaluation at 24 months. Despite promising results, the study was considered preliminary due to a small sample size (n = 84) and the lack of a confirmatory diagnosis at 36 months. Chawarska and colleagues (2014) used the same methods to predict ASD outcome at 36 months in a cohort of high-risk siblings at 18 months. The aim was to identify the individual items of the ADOS-G at 18 months that best differentiated high-risk siblings who were going to develop ASD from typically developing siblings or siblings with other developmental disorders. The combination of six behavioural features (i.e. repetitive behaviours, eye contact, intonation, gestures, giving objects and spontaneous pretend play) allowed the identification of ASD with high accuracy (83%), while poor eye contact or limited gestures alone did not provide good prognostic value for ASD. This suggests that the *interaction between (or combination of)* individual behaviours must be considered to enhance predictive value for an early identification of later ASD outcome. Prior to our study, classification of ASD from behavioural measures before 12 months has not been reported, while it would be crucial to enable early intervention. Furthermore, previous studies only looked at items from the ADOS, but did not investigate whether different measures of developmental skills and functioning can increase predictive power for ASD at an early age.

The aim of the present study was to investigate predictive longitudinal differences from 8 to 36 months between infants at low and high familial risk for autism with different developmental outcomes (typical, ASD, atypical). Further, we investigated whether we could predict ASD or atypical development at 36 months at an individual level within the HR group from data collected at 8 and 14 months. Extending the approach adopted in previous studies, we integrated measures from ASD symptoms, developmental and adaptive functioning, and we compared classifiers based on different combinations of measures to identify which combination is most predictive. We tested the hypothesis that integration of information about symptoms, developmental ability and everyday functioning can improve prediction of ASD compared to prediction from ASD-specific symptoms alone, capturing pervasiveness and addressing the high heterogeneity of ASD. Prediction was also made taking into consideration the dynamics of development by adding the change of scores between 8 and 14 months to cross-sectional measures at 8 months. This allowed us to test our second hypothesis that integration of measures from multiple time-points adds value to prediction of ASD from measures at early age compared to prediction from measures at single time-points.

## Methods

### Participants

Data presented in the current paper were collected as part of a large longitudinal study, to which 247 infants participated in one of two phases of longitudinal assessments (104 in Phase 1 and 143 in Phase 2). Data from 232 infants (161 [69.4%] high-risk siblings [HR] and 71 [30.6%] low-risk controls [LR]) were included in this study; ten infants were excluded because they did not receive an ADOS evaluation and/or a clinical outcome evaluation at 36 months; five infants were excluded because they did not attend at least one of the visits. HR infants were at increased familial risk because they had an older biological sibling with ASD, while LR controls had an older full sibling with typical development. The sample was balanced in gender (116 males and 116 females), and 85/161 HR siblings (53%) and 31/71 LR controls (44%) were males. We used imputation through expectation maximization to handle missing data (see *Supplemental Material* for details). Analyses were performed on SPSS (http://www.ibm.com/analytics/us/en/technology/spss).

### Developmental Assessments

All infants, irrespective of diagnosis and risk group, were followed longitudinally on four visits from an intake evaluation at 8 months [mean = 8.1; standard deviation, SD = 1.2] with further assessments at 14 months [mean = 14.5; SD = 1.3], 24 months [mean = 25.4; SD = 3.1] and 36 months [mean = 38.4; SD = 2.3]. At each assessment, infants were evaluated on the MSEL and VABS. Autism symptoms were assessed through the AOSI at 8 and 14 months, while the ADOS was used at 24 and 36 months. The Autism Diagnostic Interview—Revised (ADI-R; (Kim et al. 2013)), a structured parent interview, was also used to assess autism symptoms at 36 months. Experimenters were aware of infants’ risk status, but assessments were blind to clinical outcome. At the time of enrolment, none of the infants had been diagnosed with any developmental condition.

### Measures

#### Developmental Skills

Verbal and non-verbal cognitive development was measured at each visit by the MSEL (Mullen 1995), a standardized developmental measure used to assess cognitive functioning between birth and 68 months. Scores are obtained in 5 scales and 2 main functional domains: the gross motor scale (GM), and the cognitive scales. The cognitive scales are visual reception (VR), fine motor abilities (FM), receptive (RL) and expressive language (EL). The Mullen Scale provides normative scores for each specific scale (average T-score = 50, standard deviation SD = 10) and a single composite score representing general intelligence (Early Learning Composite, ELC; average standard score = 100, SD = 15). The T-scores from the five MSEL scales were included in this study.

#### Adaptive Functioning

The VABS (VABS-II) (Sparrow et al. 2005) is a semi-structured parent-report questionnaire (used at 8 and 14 months) or parent interview (used at 24 and 36 months) completed at each visit to assess infant’s adaptive behaviour in everyday settings. The items address personal and social functioning in four different domains: Communication (Comm), Daily Living Skills (DL), Socialization (Soc) and Motor Abilities (Mot). An Adaptive Behavior Composite (ABC) provides an overall index of adaptive functioning. The standard scores (mean = 100, SD = 15) from the four domains were included in this study.

#### Early ASD Symptoms

Early autism symptoms were measured at 8 and 14 months by the AOSI (Bryson et al. 2008), a semi-structured observational assessment designed to detect putative behavioural signs of autism in infants aged between 6 and 18 months. In this study a 19 item version of the AOSI was used (Brian et al. 2008), and the total score obtained from the sum of codes from the different items as an overall evaluation score was included in the analyses. The ADOS (Lord et al. 2000) was administered at 24 and 36 month but not included in our analyses. It is a standardized diagnostic instrument for the assessment of communication, social interaction, play and restricted and repetitive behaviours in children older than 18 months.

### Clinical Outcome

Expert clinical researchers reviewed all available information at 24 months (including MSEL, VABS and ADOS) and 36 months (including MSEL, VABS, ADOS and ADI-R) and assigned clinical consensus best estimate diagnosis of ASD according to ICD-10 criteria (World Health Organization 1993) to HR infants recruited in Phase 1. The same process was followed in Phase 2 and clinical consensus on ASD diagnosis was assigned according to the then published DSM-5 criteria (American Psychiatric Association 2013). To check for differences in categorisation between samples, the clinical research lead (TC) reviewed the best estimate diagnoses for the two cohorts together with the team members involved in the diagnostic decision-making, and given the lack of precision in definition in ICD-10 criteria for ‘broader ASD’ (atypical autism, PDD-unspecified, PDD-other), the broad ASD categorisation being used in both Phases was considered to be similar. Among infants who did not meet criteria for ASD, a subgroup of siblings showed atypical scores and was classified as ‘atypical’. Criteria for an atypical outcome were: ADOS and/or ADI-R above ASD threshold, and/or MSEL more than 1.5 standard deviations below average on receptive language and/or expressive language and/or early learning composite. Overall, 32/161 (19.9%) HR infants (24 males) met criteria for ASD at 36 months (*HR-ASD*); 43/161 (26.7%) HR infants (23 males) met criteria for atypical developmental (*HR-Atypical*); the remaining 86 HR infants did not meet criteria for ASD or any developmental condition (*HR-Typical*). No formal clinical diagnoses were assigned to the LR group, which was only based on risk sampling assignation, but none of them had a community clinical ASD diagnosis at 36 months. In particular, no ADI-R was administered to LR in Phase 1, who did not receive an outcome evaluation. In Phase 2, LR infants were administered the ADOS and ADI-R and received an outcome evaluation at 36 months, but none of them raised any concern for ASD or atypical development.

### Statistical Analyses: An Overview

First, four analysis groups were derived based on combined clinical outcome and risk status: *LR* (n = 71), *HR-ASD* (n = 32), *HR-Atypical* (n = 43), and *HR-Typical* (n = 86). A fractional rank based inverse normal transformation was applied to all measures and the transformed data met assumptions of normality, except for MSEL GM and RL scores, and VABS DL scores at 8 months (*p* < 0.05), AOSI total score at 14 months (*p* < 0.001), and MSEL FM scores at 36 months (*p* < 0.05). However, the statistical tests used in this study were very robust and insensitive to violations of normality. Second, to identify differences in developmental trajectories, we compared the four groups with respect to longitudinal profiles of single measures from 8 to 36 months using multilevel mixed modelling. Finally, we performed a classifier analysis on single and multiple measures from single and multiple time points to investigate whether integrated information improved prediction of ASD at pre-diagnostic age.

### Trajectory Analysis

We used measures of developmental level and adaptive functioning to characterise longitudinal profiles over four visits between 8 and 36 months. The main analysis consisted of linear mixed-effect regression, LMER, to model trajectories of each measure at group level after considering effects at the individual level. AOSI Total Score was excluded from these analyses as it was only available at two different time-points. In contrast to a more traditional approach, LMER allows to control for the variance explained by random factors without the necessity to aggregate data (Judd et al. 2012). Real age and outcome were included as fixed factors, and gender was included as a covariate, while random effects on intercept and slope were modelled on individual level. We compared linear and quadratic models on age to select the best fit for each variable based on chi-squared tests on the log-likelihood values. Then, we investigated the main effects of *outcome* and *age* (and *age*^2^ for quadratic models), and their interaction effects using Wald tests with Satterthwaite approximation for degrees of freedom. Post-hoc Tukey’s tests for multiple comparisons were performed for group comparisons and simple main effects analysis. Finally, we characterised trajectories of estimated values per different outcome groups, and 95% confidence intervals were computed via bootstrap (n = 1000 repetitions). Analyses were implemented using the *lme4* software package on R (Bates et al. 2015).

### Classifier Analysis

Classification is the process of taking some input measures (features) for a series of cases and assigning a binary label (class) to each case. In supervised learning, the classifier, which is an algorithm that implements classification using a specific set of features, is trained on a set of cases with known labels, and its predictive performance is evaluated on a separate test set with labels unknown to the classifier. In this study, we performed a supervised classifier analysis on infants using as features MSEL, VABS and AOSI scores at 8 and 14 months. The distinction made was between *HR-ASD* versus *HR-Atypical* + *HR-Typical*. In addition, the classification of *HR-ASD* + *HR-Atypical* versus *HR-Typical* was performed since the differentiation of the atypical group as a whole from typically developing infants might be useful for identifying HR siblings who would benefit from intervention. Low-risk controls were excluded from the classifier analysis since our main aim was to answer the clinically relevant question of predicting ASD outcome among HR siblings.

The algorithm chosen for classification was a least-squares support vector machine. To validate the classifier against overfitting and allow generalizability, we used 40% holdout cross-validation repeated ten times. This is a variant of *k-fold* cross-validation in which we choose the percentage of splitting between training and test sets, and the *k* number of repetitions of the learning process independently. Analyses were implemented on Matlab R2016b (MATLAB 9.1, The MathWorks Inc., Natick, MA, 2016) using the Matlab toolbox *LS-SVMlab* (http://www.esat.kuleuven.be/sista/lssvmlab). To maintain correctly evaluated predictive performance, the sample partition into training and test set was made with stratification based on outcome, so that the different sets had similar structure. Furthermore, sampling with replacement was performed on the training set to address class imbalance and avoid a wrong identification of model parameters in favour of the majority class. Model parameters were tuned via an inner cycle of 10-fold cross-validation and the tuning parameters were optimized in a Bayesian framework (Van Gestel et al. 2002). Features were z-scored before being entered into the classifier to have similar ranges of scores.

To investigate the predictive power of measures across time, we tested different classifiers using measures from different time points: 8; 14; and 8 months plus the change factor between 8 and 14 months. Then, to determine the best classifier, we computed the AUC and we evaluated each classifier performance via sensitivity, specificity, accuracy, negative predictive value (NPV: true negative over negative predicted cases) and positive predictive value (PPV: true positive over positive predicted cases). 95% confidence intervals (CI) for each metric were computed to improve reliability of the obtained estimates using bootstrap with n = 1000 repetitions for each cross-validation fold, then averaging over folds. To test whether classification accuracy was significantly better than chance level, we computed the *p-value* of AUC for each classifier through a shuffle test. Labels in the test set were randomly shuffled, and pre-trained classifiers were used for prediction on the test set. This procedure was repeated n = 1000 times for each cross-validation fold (n = 10,000 total repetitions) to estimate the null distribution of AUC and test whether classifiers perform significantly better than random. Then, a nonparametric Friedman test was performed on the AUC of different classifiers at each time point separately to test whether accuracy differed using different measures from the same time point as features. When the Friedman test was significant, we performed post-hoc paired Wilcoxon tests between the classifier with highest AUC and other classifiers. The Bonferroni correction was used to account for biasing effects due to multiple comparisons. The same method was used to test whether the same measures at different time points provided different predictive accuracy. Finally, the paired Wilcoxon test was used to test whether predictive accuracy of the best classifiers at different time points was significantly different within the same classification (*HR-ASD vs. HR-Typical + HR-Atypical* or *HR-ASD* + *HR-Atypical vs. HR-Typical*), and whether predictive accuracy of the best classifiers at the same time points was significantly different between the two different classifications. This allowed us to assess differences in predictive power for ASD at different time points, and to compare predictive accuracy for ASD versus broader atypicality. Classifier comparisons were performed on SPSS (http://www.ibm.com/analytics/us/en/technology/spss).

## Results

### Participant Characteristics

Global descriptive statistics are summarized in Table 1. There was a significant difference of gender per clinical outcome, with more males receiving an ASD diagnosis at 36 months than females (odd ratio for HR males vs. females developing into ASD OR = 3.52 [CI: 1.51–8.22], *p* < 0.005). Outcome groups did not differ from each other in age at any visit.

**Table 1:** Demographic data for high-risk and low-risk groups by 36-month clinical outcome

	Overall	High-Risk (n = 161)			Low-Risk (n = 71)
		ASD (n = 32)	Atypical (n = 43)	Typical (n = 86)	
Gender	n	n	n	n	n
Male	116	24	23	38	31
Female	116	8	20	48	40

	mean (SD)	mean (SD)	mean (SD)	mean (SD)	mean (SD)
Age					
8 m	8.13 (1.22)	8.03 (1.12)	8.33 (1.06)	8.24 (1.21)	7.92 (1.35)
14 m	14.48 (1.27)	14.50 (1.32)	14.56 (1.20)	14.58 (1.29)	14.31 (1.26)
24 m	25.39 (3.06)	24.84 (1.63)	26.40 (4.25)	25.72 (2.31)	24.63 (3.30)
36 m	38.39 (2.32)	38.06 (1.90)	38.19 (2.05)	38.62 (2.29)	38.39 (2.69)
MSEL					
GM 8 m	47.24 (10.68)	43.84 (11.37)	45.07 (12.57)	47.31 (10.48)	50.00 (8.68)
GM 14 m	49.57 (14.79)	45.59 (14.46)	47.60 (13.01)	50.87 (14.97)	50.97 (15.54)
GM 24 m	51.54 (11.50)	46.95 (14.02)	49.46 (10.60)	50.91 (11.39)	55.65 (9.72)
FM 8 m	55.10 (12.41)	48.53 (12.91)	52.00 (12.78)	56.77 (12.71)	57.92 (10.17)
FM 14 m	55.69 (9.95)	50.50 (11.63)	52.63 (11.07)	56.50 (8.43)	58.89 (8.83)
FM 24 m	48.77 (10.67)	44.64 (11.55)	45.17 (13.30)	48.49 (8.95)	53.15 (8.83)
FM 36 m	51.40 (16.39)	39.84 (16.09)	43.30 (16.01)	54.34 (14.80)	57.96 (14.02)
VR 8 m	53.81 (11.07)	51.59 (10.46)	50.49 (11.16)	54.35 (11.75)	56.17 (9.95)
VR 14 m	49.99 (9.98)	45.09 (9.31)	48.53 (9.56)	48.95 (9.96)	54.35 (9.05)
VR 24 m	53.62 (12.85)	47.30 (13.08)	46.92 (13.62)	55.18 (10.59)	58.65 (12.14)
VR 36 m	56.83 (13.82)	49.29 (17.54)	49.47 (15.04)	60.51 (10.88)	60.21 (11.29)
RL 8 m	47.56 (10.17)	43.35 (12.50)	46.05 (8.70)	49.90 (10.08)	47.55 (9.33)
RL 14 m	42.64 (11.90)	36.19 (9.05)	40.19 (10.73)	43.68 (12.31)	45.77 (12.01)
RL 24 m	52.17 (12.99)	41.71 (15.47)	46.92 (13.17)	53.26 (10.74)	58.75 (9.71)
RL 36 m	52.53 (12.85)	43.47 (17.71)	43.37 (13.04)	55.80 (8.37)	58.21 (9.15)
EL 8 m	51.05 (10.21)	50.08 (11.89)	51.26 (11.00)	50.52 (10.14)	52.01 (9.07)
EL 14 m	47.77 (10.66)	42.13 (11.44)	44.98 (10.90)	49.64 (9.93)	49.75 (9.93)
EL 24 m	51.60 (12.93)	46.23 (15.30)	47.72 (13.20)	50.70 (11.49)	57.46 (11.18)
EL 36 m	53.95 (12.86)	43.28 (16.14)	45.20 (12.31)	57.76 (9.33)	59.39 (9.38)
VABS					
Comm 8 m	95.73 (15.88)	90.19 (15.33)	89.53 (17.12)	96.35 (15.71)	101.23 (13.58)
Comm 14 m	96.59 (13.30)	86.04 (14.28)	93.17 (15.85)	98.35 (11.63)	101.29 (9.68)
Comm 24 m	103.31 (12.73)	94.47 (15.06)	98.41 (10.78)	104.31 (10.66)	109.06 (11.88)
Comm 36 m	101.12 (14.28)	88.96 (18.19)	93.18 (14.03)	103.26 (10.24)	108.82 (10.42)
DL 8 m	99.95 (13.49)	93.56 (15.28)	98.98 (11.75)	101.24 (13.14)	101.87 (13.39)
DL 14 m	95.17 (12.99)	85.63 (13.39)	93.38 (13.38)	96.58 (12.62)	98.86 (10.80)
DL 24 m	105.48 (12.48)	97.88 (13.95)	101.49 (13.50)	107.55 (11.09)	108.83 (10.80)
DL 36 m	103.06 (13.03)	88.36 (18.03)	97.74 (12.64)	106.63 (8.84)	108.60 (7.91)
Mot 8 m	89.65 (16.21)	85.13 (16.71)	80.65 (15.66)	90.73 (15.88)	95.84 (13.80)
Mot 14 m	100.33 (12.84)	98.06 (14.27)	95.73 (15.10)	100.40 (11.59)	104.04 (11.15)
Mot 24 m	99.97 (10.66)	98.19 (12.49)	96.51 (11.83)	99.50 (10.09)	103.44 (8.77)
Mot 36 m	93.66 (12.23)	84.66 (13.25)	86.47 (10.48)	95.79 (10.35)	99.49 (10.54)
Soc 8 m	99.84 (12.72)	96.97 (15.66)	97.93 (11.52)	99.62 (11.85)	102.55 (12.71)
Soc 14 m	97.77 (11.66)	91.40 (11.78)	96.53 (12.66)	98.44 (11.06)	100.58 (10.71)
Soc 24 m	100.72 (11.46)	88.91 (11.68)	97.33 (10.65)	101.99 (8.21)	106.56 (10.74)
Soc 36 m	97.78 (12.89)	79.64 (12.66)	92.43 (11.75)	100.93 (8.68)	105.38 (8.00)
AOSI					
8 m	8.34 (4.86)	10.66 (5.71)	9.47 (4.86)	8.51 (4.66)	6.39 (3.96)
14 m	5.10 (4.34)	7.59 (4.42)	7.05 (4.94)	4.48 (3.98)	3.56 (3.41)
ADOS					
24 m	2.67 (4.16)	6.59 (7.02)	2.72 (3.19)	2.13 (3.15)	1.52 (2.91)
36 m	6.38 (5.18)	11.19 (6.38)	10.28 (5.92)	3.63 (2.37)	5.17 (3.45)

### Longitudinal Characterization of Development

**Fig. 1:**
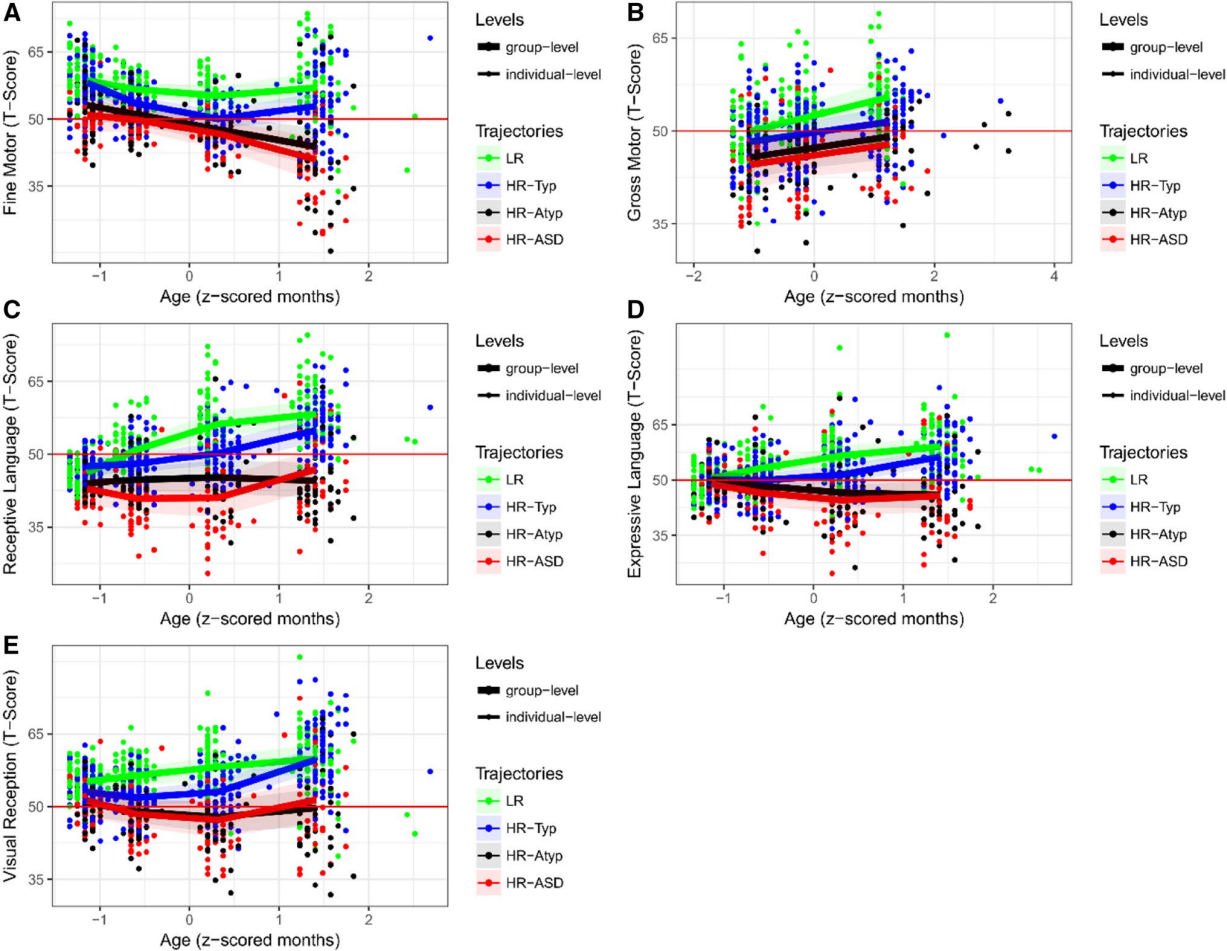
Developmental trajectories of estimated means for MSEL measures by clinical outcome groups. This figure shows the longitudinal trajectory of scores per outcome groups (LR, *HR-Typical, HR-Atypical, HR-ASD*) obtained through multilevel mixed modelling for each scale of the MSEL. The developmental trajectories are built on four time-points, one for each visit, which are approximately: 8; 14; 24; 36 months. 95% bootstrap confidence interval on group trajectories is shown as shaded area. Individual scores are also shown (points) with different colours by outcome group. The average normative score is shown by the red line. *MSEL* Mullen Scales of Early Learning, *LR* low-risk controls, *HR* high-risk siblings

**Fig. 2:**
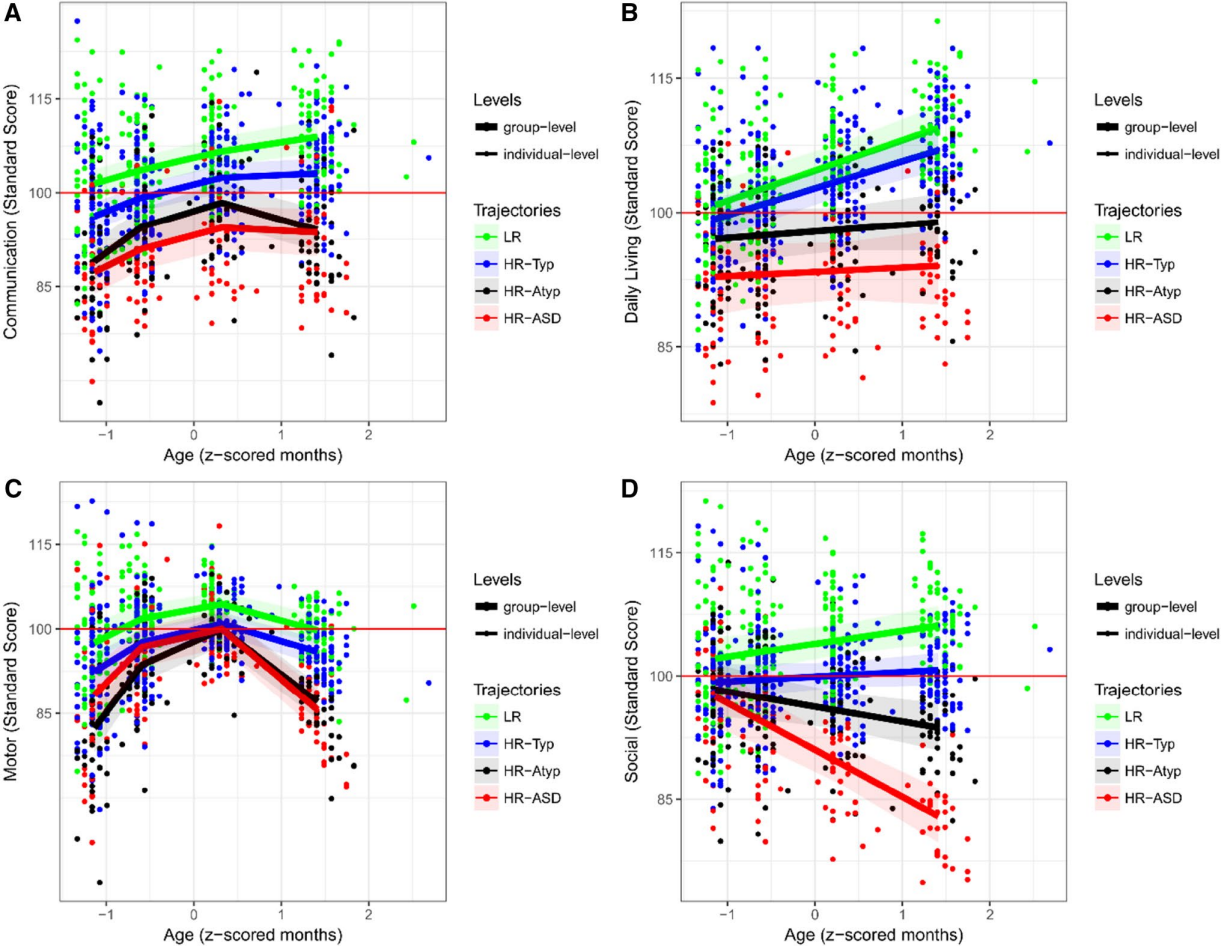
Developmental trajectories of estimated means for VABS measures by clinical outcome groups. This figure shows the longitudinal trajectory of scores per outcome groups (LR, *HR-Typical, HR-Atypical, HR-ASD*) obtained through multilevel mixed modelling for each scale of the VABS. The developmental trajectories are built on four time-points, one for each visit, which are approximately: 8; 14; 24; 36 months. 95% bootstrap confidence interval on group trajectories is shown as shaded area. Individual scores are also shown (points) with different colours by outcome group. The average normative score is shown by the red line. *VABS* Vineland Adaptive Behavior Scales, *LR* low-risk controls, *HR* high-risk siblings

Developmental trajectories were characterised for group contrasts (*LR* vs. *HR-Typical* vs. *HR-Atypical* vs. *HR-ASD*). Figure 1 shows trajectories of MSEL scores, and Fig. 2 shows trajectories of VABS scores. Detailed statistics can be found in Supplemental Material. Since both MSEL and VABS scores were standard scores normed for age, increasing or decreasing developmental trajectories should be interpreted as individuals developing skills either more rapidly or more slowly than expected based on age-appropriate norms. Moreover, the main effect of age must be examined taking into consideration the interaction with outcome.

In terms of trajectories of MSEL scores, we found significant main effect of outcome across all scores (*p*s < 0.001, except for GM: p < 0.005), and significant main effect of age across all scores (*p*s < 0.001) except for EL scores. Specifically, all measures showed quadratic growth (Chi-squared *p* < 0.001) except for gross motor scores, which had linear growth over time. Furthermore, the interaction effect between outcome and age was statistically significant for fine motor scores (*p* < 0.005), receptive language (*p* < 0.005), expressive language (*p* < 0.001), and visual reception scores (*p* < 0.05). Tukey’s post-hoc tests showed that the main effect of outcome in gross motor scores was mainly driven by a differentiation between *LR* and *HR-Atypical* and *HR-ASD* (respectively *p* = 0.018 and *p* = 0.007). Similarly, simple main effect analysis showed that the interaction of outcome and age in the other measures was mainly driven by slower increases or decreases in developmental trajectories of *HR-Atypical* and *HR-ASD* compared to LR and *HR-Typical*, leading to an increased differentiation over time which became significant around 14 months. Fine motor scores were the only exceptions, showing significant differentiation between *LR* and *HR-ASD* already by 8 months (*p* < 0.05).

Across VABS scores, linear growth models were the best fit for daily living and social scores, while communication and motor scores showed quadratic growth. We observed significant main effect of age across all scores (*p* < 0.001), and significant main effect of outcome in communication, daily living, and social scores (*p* < 0.001). Overall, results showed an increasing gradient of scores from *HR-ASD* to *LR*, with significant differences between *LR* and *HR-ASD* across all scores except for motor scores (Comm and Soc: *p* < 0.001; DL: *p* < 0.005); between *LR* and *HR-Atypical* across all scores (Comm: *p* < 0.005; DL: *p* < 0.05; Soc: *p* < 0.001; Mot: marginal *p* = 0.08); between *HR-Typical* and *HR-Atypical* in daily living (*p* < 0.05) and social scores (marginal significance: *p* = 0.052); and between *HR-Typical* and *HR-ASD* across all measures except for motor scores (Soc and DL: *p* < 0.001; Comm: *p* < 0.005). Differences between LR and *HR-Typical* were significant in social scores (*p* < 0.05) and marginal in communication scores (*p* = 0.07), while *HR-Atypical* only had significantly higher social scores than *HR-ASD* (*p* = 0.018). Yet, across all scores the interaction effect between outcome and age was statistically significant (Comm: *outcome* × *age*^2^, *p* = 0.017; DL: *outcome* × *age, p* = 0.024; Soc: *outcome* × *age, p* < 0.001; Mot: *outcome* × *age*^2^, *p* < 0.001). Thus, we performed an analysis of simple main effects. The difference between *LR* and *HR-Typical* and *HR-Atypical* and *HR-ASD* was clear from 8 months in communication, daily living and motor scores. The interaction effect in motor scores was mainly driven by a rapid decrease of scores for *HR-Atypical* and *HR-ASD* between 24 and 36 months, while the initial delay of *HR-Atypical* with respect to *LR* and *HR-Typical* was recovered by 24 months. Similarly, interaction in communication was mainly due to an increase in scores for *LR* and *HR-Typical*, and a decrease for *HR-Atypical* and *HR-ASD* between 24 and 36 months. The interaction in daily living skills was due to an increase of scores over time for *LR* and *HR-Typical*, while *HR-Atypical* and *HR-ASD* were stable below average. Finally, divergent developmental trajectories were clearly visible in social scores: from a steady decrease over time for *HR-ASD* to a slight increase for *LR*, reaching a complete group differentiation at 36 months.

### Predicting ASD from Different Instruments and Functional Domains

**Fig. 3:**
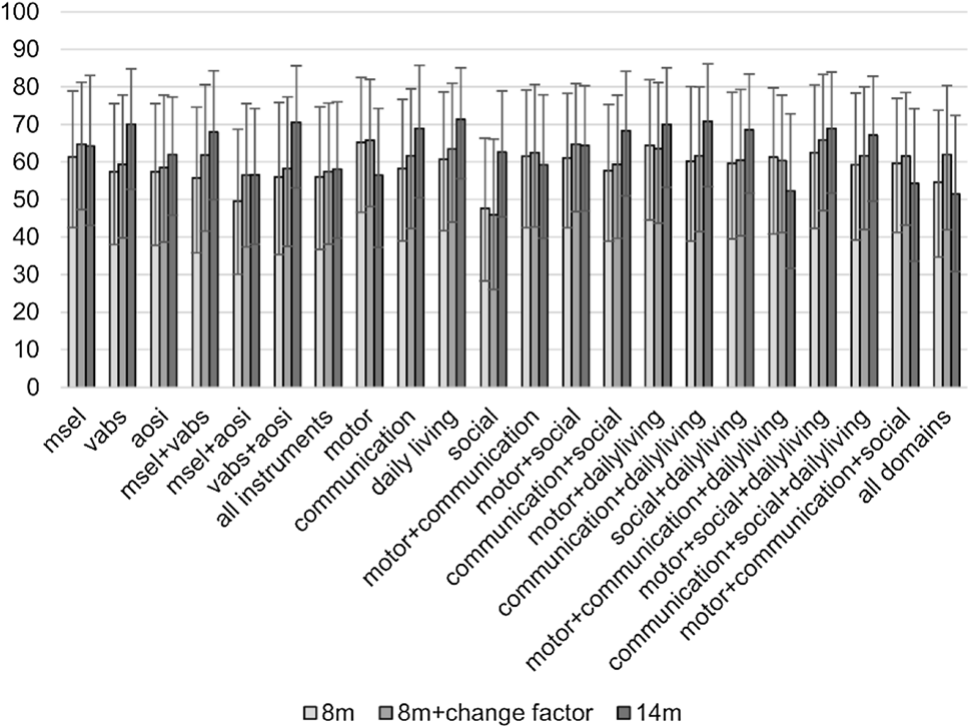
Prediction of ASD clinical outcome at 36m: AUC. In this figure the area under the curve (AUC) is reported for different classifiers based on behavioural measures (MSEL, VABS and AOSI) and their combination at different time-points (8 months, 8 months + change factor, 14 months). The classification made is between high-risk infants who are going to develop ASD at 36 m, and high-risk infants with typical and atypical (but not ASD) outcome at 36 m. The change factor is computed as the difference between measures at 14 and 8 months over the age difference between the two visits. The 95% confidence interval is also reported for each classifier. *AUC* area under the curve, *MSEL* Mullen Scales of Early Learning (5 scores), *VABS* Vineland Adaptive Behavior Scales (4 scores); *AOSI* Autism Observation Scale for Infants, in this study we considered the total score

Next, we classified *HR-ASD* outcome as different from *HR-Atypical* and *HR-Typical* outcome by the integration of data from different instruments and different functional domains at pre-diagnostic ages (i.e. 8 and 14 months), and assessed the added value of data integration when compared to prediction using data from single functional domains or a single instrument. Figure 3 shows the AUC for the different classifiers.

Considering all classifiers, AUC ranged between 48 and 65% for prediction at 8 months, when all classifiers had predictive performance at chance level. At 14 months, VABS daily living scores showed the best predictive performance (AUC = 71.3% [CI 55.6–85.1]; sensitivity = 79.6% [CI 55.2–96.6], specificity = 52.2% [CI 38.7–65.7], accuracy = 57.5% [CI 45.3–69.2], PPV = 28.5% [CI 14.1–43.8], NPV = 91.5% [CI 80.3–98.7]). This was significantly different from chance level, but not from the classifiers with highest AUC at earlier time points, while differences in performance from other classifiers at 14 months missed significance after Bonferroni correction for multiple comparisons.

To assess the added value of data from multiple time points, we tested predictive performance of classifiers built from cross-sectional scores plus the change factor between time points. As with prediction at 8 months, integrated measures from 8 and 14 months predicted ASD at chance level. However, predictive accuracy improved in comparison to measures at 8 months for the following classifiers: the integration of Mullen and Vineland scores (*z* = − 2.80, *p* = 0.005); communication scores (*z* = − 2.70, *p* = 0.007); the integration of motor, social and daily living scores (*z* = − 2.81, *p* = 0.005); daily living scores (marginally; *z* = − 2.40, *p* = 0.017); the integration of communication, social and daily living scores (marginally; *z* = − 2.40, *p* = 0.017); and the integration of Vineland scores and the AOSI total score (trend level; *z* = − 2.10, *p* = 0.036). Thus, the rate of developmental change improved predictive accuracy for ASD but prediction was still at chance level. Detailed statistics can be found in the Supplemental Material.

**Fig. 4:**
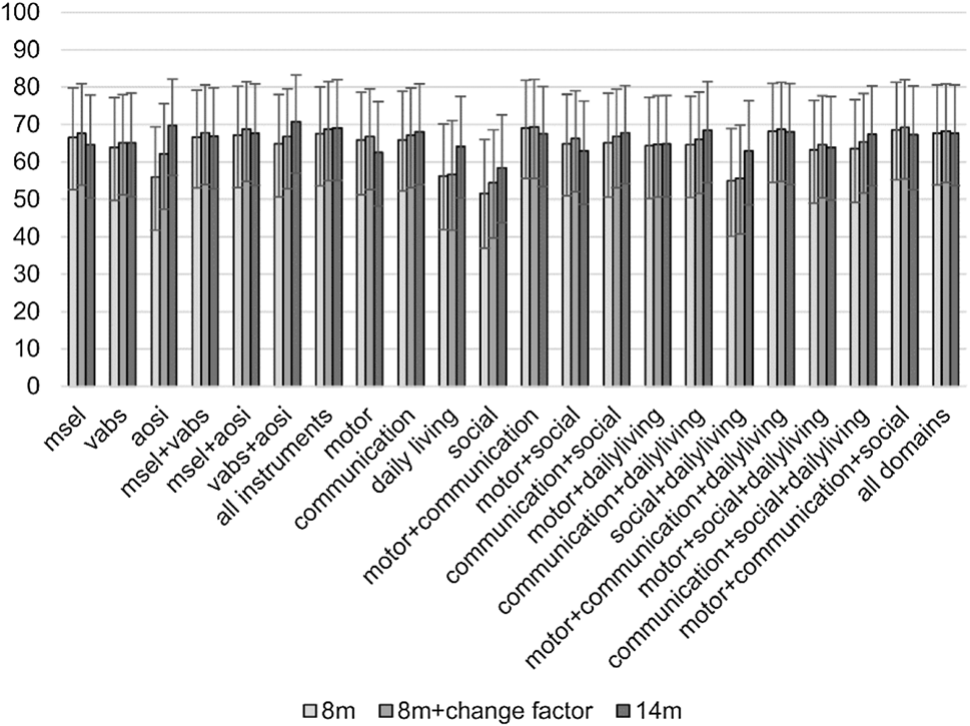
Prediction of atypical clinical outcome (including ASD) at 36 m: AUC. In this figure the AUC is reported for different classifiers based on behavioural measures (MSEL, VABS and AOSI) and their combination at different time-points (8 months, 8 months + change factor, 14 months). The classification made is between high-risk infants with atypical development (including an ASD diagnosis at 36 m), and high-risk infants with typical outcome at 36 m. The change factor is computed as the difference between measures at 14 and 8 months over the age difference between the two visits. The 95% confidence interval is also reported for each classifier. *AUC* area under the curve, *MSEL* Mullen Scales of Early Learning (5 scores), *VABS* Vineland Adaptive Behavior Scales (4 scores); *AOSI* Autism Observation Scale for Infants, in this study we considered the total score

Next, we predicted *HR-ASD* and *HR-Atypical* outcome together as different from *HR-Typical*. The AUC for the different classifiers is shown in Fig. 4, while Table 2 shows metrics of the best performing classifiers at each time point for each classification problem. Details on classifier performance at different time points can be found in the Supplemental Material.

**Table 2:** Best classifiers at each time-point for the two different classifications

Classification	Classifier	*p*	AUC (%)	Sensitivity (%)	Specificity (%)	Accuracy (%)	PPV (%)	NPV (%)
8 months								
*HR-ASD vs*. (*HR-Atypical* + *HR-Typical*)	Motor scores	0.11	65.1 (46.6, 82.5)	64.2 (38.1, 89.7)	65.6 (52.7, 78.0)	65.3 (53.6, 76.6)	30.8 (13.6, 49.6)	88.7 (77.9, 97.8)
(*HR-ASD* + *HR-Atypical*) *vs. HR-Typical*	Motor + communication scores	0.01^*^	69.2 (55.6, 81.4)	68.8 (51.6, 85.0)	64.4 (47.8, 79.7)	66.4 (55.2, 77.7)	62.8 (46.0, 79.2)	70.3 (53.5, 85.8)
8 months + change factor								
*HR-ASD vs*. (*HR-Atypical + HR-Typical*)	Motor + social + daily living scores	0.10	65.9 (47.0, 83.3)	61.9 (33.4, 88.1)	65.0 (51.5, 77.2)	64.4 (52.5, 75.6)	30.0 (12.7, 47.9)	87.6 (76.1, 96.7)
(*HR-ASD + HR-Atypical*) *vs. HR-Typical*	Motor + communication scores	0.01*	69.4 (55.6, 82.1)	70.5 (52.9, 86.2)	67.8 (51.7, 82.8)	69.1 (57.5, 80.2)	65.9 (48.7, 81.9)	72.5 (56.4, 87.2)
14 months								
*HR-ASD vs*. (*HR-Atypical + HR-Typical*)	Daily living score	0.03*	71.3 (55.6, 85.1)	79.6 (55.2, 96.6)	52.2 (38.7, 65.7)	57.5 (45.3, 69.2)	28.5 (14.1, 43.8)	91.5 (80.3, 98.7)
(*HR-ASD + HR-Atypical*) *vs. HR-Typical*	VABS scores + AOSI total score	0.01^*^	70.8 (57.1, 83.2)	60.7 (43.2, 78.3)	67.5 (51.0, 82.7)	64.4 (52.0, 75.9)	62.1 (43.5, 79.5)	66.4 (50.2, 81.6)

Performance metrics for the classifiers chosen as best (based on having the highest AUC) at different age for classifying HR atypically developing siblings (including those who later develop ASD) from their typically developing peers (*HR-ASD* + *HR-Atypical vs. HR-Typical*), and HR sibling who later develop ASD from those who do not (*HR-ASD vs. HR-Atypical* + *HR-Typical*). The significance of classification AUC was determined by permutation test, the resulting *p-values* are reported. Prediction was considered significant if *p* < 0.05 (marked as ^*^). 95% confidence interval is reported in parentheses. All metrics are reported as mean (lower level CI, upper level CI).

*AUC* area under the curve, *PPV* positive predictive power, *NPV* negative predictive power, *MSEL* Mullen Scales of Early Learning (5 scores), *VABS* Vineland Adaptive Behavior Scales (4 scores); *AOSI* Autism Observation Scale for Infants, in this study we considered the total score

Considering all classifiers, AUC ranged between 52 and 69% using measures at 8 months, and similarly using measures at 14 months or from integrated time points. By integrating measures from 8 and 14 months, predictive accuracy increased with respect to 8 months for: AOSI total score (*z* = − 2.70, *p* = 0.007 with α = 0.017); the integration of Vineland scores and AOSI total score (*z* = − 2.60, *p* = 0.009); and motor scores (trend level; *z* = − 2.10, *p* = 0.037) (see Supplemental Material for details). The best classifier for prediction of *HR-ASD* plus *HR-Atypical* integrated VABS scores and AOSI total scores at 14 months (AUC = 71.8% [CI 58.3–83.7]; sensitivity = 62.2% [CI 44.0–79.0], specificity = 69.5% [CI 53.1–84.6], accuracy = 66.1% [CI 54.4–77.0], PPV = 63.9% [CI 45.7–81.3], NPV = 68.2% [CI 51.7–82.8]). As with classification of ASD, performance of the best classifier at 14 months did not differ from the classifiers with highest AUC at earlier time points, or the other classifiers at 14 months after Bonferroni correction.

Finally, we tested differences in accuracy of classifiers for the two classification problems to examine whether predictive power was different for ASD and broader atypical development and found no statistically significant difference (see Supplemental Material).

## Discussion

This is the first study in which integrated standardized behavioural measures in infancy were used to characterise developmental trajectories at group-level and to individually classify HR siblings who later develop ASD from their typically and atypically developing peers. Our hypotheses were that integration of information from multiple functional domains and multiple time points would improve early prediction of ASD compared to prediction from a single domain or a single time point. Our main findings were: (1) clear but small size group effects for Mullen and Vineland scores between LR, *HR-ASD, HR-Atypical* and *HR-Typical* outcome groups at 8 and 14 months, and larger group effects at 24 and 36 months; (2) individual prediction of ASD from non-ASD outcome at chance level at 8 months, but at moderate and above chance level (AUC = 71.3%) at 14 months; (3) individual prediction of broader atypical development from typical outcome with moderate AUC at 8 and 14 months (approximately 70%); (4) added value of combined measures for prediction of broader atypical from typical outcome, but not for prediction of ASD from non-ASD outcome; and (5) added value of combined time points to prediction for some, but not all measures.

### Developmental Trajectories at Group Level

Differences in development between LR and *HR-Typical* versus *HR-Atypical* and *HR-ASD* drove the differentiation of outcome groups over time. Specifically, developmental trajectories of *LR* and *HR-Typical* infants were either stable or increasing across all scores, showing normative or above average development in respect to age-appropriate norms. In contrast, developmental trajectories of *HR-Atypical* and *HR-ASD* siblings were stable or decreasing across all scores, indicating that those infants tend to fall behind age-appropriate norms during development. This was particularly true for VABS social scores. Furthermore, we observed a gradient of scores across groups in MSEL motor scores and VABS communication, daily living and social scores between 8 and 36 months. Specifically, LR scores were higher than *HR-Typical* scores, which were higher than *HR-Atypical* scores, which were higher than *HR-ASD*. In contrast, VABS motor and MSEL visual reception and language scores showed overlapping or crossing trajectories for *HR-Atypical* and *HR-ASD*.

We observed differences between groups from 8 months, supporting and extending results from previous studies which showed differences between *HR-ASD* and *LR*, or *HR-ASD* and *HR-Typical* on several measures at 8 months (Zwaigenbaum et al. 2005; Gammer et al. 2015). In particular, delays in high-risk infants who later developed ASD tended to start in the motor domain at 8 months and extend to the social domain by 14 months. This confirms and extends previous findings to high-risk siblings who received a clinical outcome evaluation at 36 months (Estes et al. 2015; Kolesnik et al. 2017). However, we found differences between *HR-ASD* and *HR-Typical* on Mullen receptive but not expressive language scores, and between LR and *HR-ASD* or *HR-Atypical* on Mullen language scores and Vineland communication scores already at 8 months. These differences in communication skills were detected earlier than previously reported (Barbaro and Dissanayake 2012; Chawarska et al. 2009). Further work is needed to understand whether the lack of group differentiation on social scores at 8 months is due to the inability of current tools to capture ASD-related manifestations on social skills at early age, or whether it reflects the developmental pathway of ASD. In particular, more granular assessments are needed to characterise developmental trajectories with greater precision and to better capture the dynamics of development.

### Individual Classification of HR-ASD from HR-Typical plus HR-Atypical

Next, we moved from group comparisons to individual prediction: our aim was to test whether it was possible to reduce the age at which individual prediction of ASD is possible, and to improve predictive power for ASD using standardized measures. Prior to this study, predictive power of Mullen, Vineland and AOSI scores had not been tested with respect to individual ASD outcome, although these instruments are largely used for clinical evaluation. On the other hand, previous studies have used ADOS scores to classify ASD (Chawarska et al. 2014; Macari et al. 2012; Wall et al. 2012).

To improve predictive power and reduce the age of individual prediction for ASD from behavioural measures, we specifically focused on data from 8 and 14 months, as previous studies classified ASD from ADOS at 18 months (Chawarska et al. 2014). Furthermore, we used different combinations of standardized behavioural measures as predictors, since neuroimaging studies had already shown higher predictive power for ASD from integrated data than data from single modalities (Libero et al. 2015; Lombardo et al. 2015). However, our results showed that the integration of different measures did not improve prediction of ASD at 8 months, which remained at chance level. This might be explained by the heterogeneity of the behavioural phenotype linked to later development of ASD in the first year of life, when behavioural atypicalities are subtle and possibly transient. ASD might not be defined as a single category in behaviour before the second year of life, when the defining behaviours generally unfold, explaining poor predictive power of our data.

We also attempted to exploit information from early developmental trajectories as additional information for classification by adding the change factor between 8 and 14 months to cross-sectional scores at 8 months. The rate of development added significant value for prediction to classifiers focused on communication skills; VABS plus MSEL scores; and the integration of motor, social and daily living scores. These results highlight the dynamical changes in development between 8 and 14 months which are relevant to ASD. However, prediction was still not different from chance level, suggesting that prediction of ASD probably depends more on the level of development and functioning rather than the rate of change. One route to improving classifiers would be to have greater density of data collection between 8 and 14 months to capture developmental dynamics with greater precision. Predictive value was not improved by adding trajectory information to classifiers using information about social skills; the integration of motor and daily living scores; and the integration of motor, communication and daily living scores. For these cases, it is possible that the change factor made the binary separation between classes more difficult by adding heterogeneity due to intra-individual heterogeneity in developmental trajectory. In fact, we found higher intra-individual heterogeneity on fine motor, communication, social, and AOSI scores in *HR-ASD* than other siblings (see Supplemental Material).

The Vineland daily living scores at 14 months provided the highest predictive power for ASD (71.3% AUC). Impairments in daily living skills, such as being careful around hot objects or following household rules, are common in children with ASD (Liss et al. 2001; Perry et al. 2009). However, their investigation is usually underestimated in very young infants because they are difficult to assess at an early age, when parents usually perform tasks for their children. Thus, it is novel to find daily living scores as best predictors for ASD at 14 months. Yet, previous studies have shown that symptom severity in young children with ASD can predict daily living skills (Perry et al. 2009), and our results show that daily living skills can be affected as soon as (or even before) clinical symptoms begin to emerge. In fact, impairments in daily living skills might reflect the accumulation of more subtle impairments in other domains, like social-communication and motor domains (Green and Carter 2014; Jasmin et al. 2009), since they require the ability to understand requests and tasks, and to perform the task itself. As a result, more complex actions measured by the daily living skills scale might enlarge the differentiation between infants who later develop ASD and those who do not. However, caution is required because differences in predictive performance from integrated measures (such as communication and daily living scores, motor and daily living scores, or Vineland and AOSI scores) failed to reach statistical significance, and differences from other classifiers were only marginally significant after Bonferroni correction. Nevertheless, it is better in practice to have a simpler predictor and our results suggest that it might be sufficient to assess daily living skills at 14 months.

In summary, despite clear group differences at various levels, individual prediction of ASD using different measures at different time points was still far from optimal. In fact, although the AUC was moderate, our most successful classifier had a much lower PPV than NPV (respectively 28.5 and 91.5%), which means that it was more accurate at predicting infants who will not develop ASD. This might be explained by the prevalence of positives (20% of *HR-ASD*), since low incidence generally reduces PPV, and the measures included in this study, which are tuned to pick up the abilities that define ‘typicality’. However, prediction of infants who are going to develop typically in all likelihood is still very useful to allay any concern. Further work is needed to allow a more accurate prediction of the minority class, for instance including data more specific to ASD. In fact, although AOSI focuses on the assessment of ASD symptoms, behaviours like shyness might be confounding. Thus, moderate classifier performance might link to critical missing variables for prediction of ASD, such as measures of home environment, social attention, or changes in the brain. Furthermore, moderate predictive accuracy might be explained by the high inter-individual variability in clinical symptoms and developmental problems in ASD. Converging evidence suggests the presence of different subgroups within infants who later develop ASD, and the heterogeneity of early developmental pathways to ASD (Jones et al. 2014; Landa et al. 2013). Measures included in this study might not be able to separate all individuals from different subgroups developing ASD from siblings who do not; other methodological approaches might be used to identify patterns of behaviours predicting ASD specific to the different subgroups. Previous studies already attempted to address heterogeneity by finding separate predictive patterns of symptoms and predicted ASD outcome at 24 months with higher accuracy than the present study (Chawarska et al. 2014; Macari et al. 2012). However, we focused on younger age points to predict outcome at 36 months.

### Individual Classification of HR-ASD plus HR-Atypical from HR-Typical

We used the same classification approach to predict broader atypical development in high-risk siblings. The integration of motor and communication scores from MSEL and VABS allowed classification with moderate accuracy at 8 months (AUC = 69.2%), and at combined 8 and 14 months (AUC = 69.4%). Differences in AUC between different classifiers (e.g. VABS + AOSI vs. VABS alone) suggested that data integration improved predictive performance, though this was only marginally significant after correcting for multiple comparisons. While delays in motor skills have been previously documented in the first year of life (Leonard et al. 2014; Libertus et al. 2014), the improved predictive accuracy of integrated communication and motor scores is in contrast with previous findings supporting the emergence of ASD in the sensorimotor domain before 12 months, and moving only later to the social-communication domain (Estes et al. 2015; Kolesnik et al. 2017). However, this might be explained by the inclusion of siblings with early emerging language delays, but not ASD, when classification of ASD is extended to classification of broader atypicality. Nevertheless, it is in line with our results at group level, which showed differentiation between *HR-ASD* and *HR-Typical* on receptive language already at 8 months.

At 14 months, the integration of VABS and AOSI scores showed the highest predictive accuracy (AUC = 70.8%), although not statistically significantly different from AOSI alone (AUC = 69.8%). Thus, although measures of ASD symptoms seem to retain most of predictive power when taken alone, classifying correctly 70 out of 100 infants, the interplay between symptoms and adaptive functioning improved prediction of broader atypical outcome by classifying correctly one more infant. The integration of measures from 8 and 14 months also improved predictive accuracy over 8-month data alone for classifiers using AOSI and Vineland plus AOSI scores, in line with previous studies showing an increase in predictive power of AOSI scores in the second year of life (Gammer et al. 2015). Thus, the rate of emergence of symptoms and the interplay with everyday functioning at the end of the first year of life might be relevant to the development of atypical vs. typical outcome. However, it did not provide additional predictive power to measures at 14 months alone.

### Strengths and Limitations

This study extends previous high-risk studies on early markers for ASD by (1) integrating information from multiple measures and multiple time-points; (2) testing models for individual classification, which is a fundamental issue for clinical practice; (3) focusing on prediction at younger age points. We used a mixed-gender sample for classification and observed a significant difference on gender per clinical outcome, with more males receiving an ASD diagnosis at 36 months than females. Yet, the addition of gender as a feature for classifiers did not significantly improve AUC, except for prediction of ASD from Vineland social scores at 8 months (Bonferroni corrected *p* = 0.02, *z* = − 3.3), and AOSI total scores at 14 months (Bonferroni corrected *p* = 0.01, *z* = − 3.4). Further work should investigate specific differences linked to gender in predictive power for ASD of behavioural and developmental measures. Although we had access to a reasonably large sample, statistical power still remains a limitation to this study. Statistical significance of differences in performance between classifiers with highest AUC and other classifiers at the same time point did not survive Bonferroni correction. Therefore, we need to increase statistical power and results need replication in larger samples. Furthermore, the *HR-Atypical* group needs careful interpretation due to the high variability of individuals included in this group, which was more instrument-defined than clinically based.

### Future Directions

Despite clear differences at group level and moderately high predictive accuracy, individual prediction still needs to be improved. Our results provide further evidence to the high inter-individual and intra-individual heterogeneity of ASD, which makes difficult to predict the later development of the disorder at an early age. Further investigation is needed to understand the interplay of different domains in the first years of life leading to an ASD outcome. It is also possible that the ASD behavioural phenotype simply does not exist as a definable category before 2 years of age. The exploratory investigation of heterogeneity of ASD development in a bottom-up approach through latent class analysis can provide better insight into the different developmental trajectories leading to ASD, and a data driven approach might be used to discover new categories than those currently used for classification. Furthermore, the combination of measures from different domains can be extended to include more biological data (e.g. genetics and epigenetics, EEG and ERP, or functional MRI data) and measures of environmental experience (e.g. parent behaviour or socioeconomic status) to provide a more complete picture of the developmental status of the infant. Future work must also investigate generalizability of predictive classifiers to provide a useful tool for clinical practice.

## Electronic Supplementary material

Below is the link to the electronic supplementary material.

Supplementary material 1 (DOCX 76 KB)
