# Supplementary material for: Prediction of Autism at 3 Years from Behavioural and Developmental Measures in High-Risk Infants: A Longitudinal Cross-Domain Classifier Analysis
Source: J Autism Dev Disord. 2018 Feb 16;48(7):2418–33. doi: 10.1007/s10803-018-3509-x (PMC5996007; doi:10.1007/s10803-018-3509-x)
Supplement: Supplementary file 1 — Supplementary material 1 (DOCX 76 KB) [file 10803_2018_3509_MOESM1_ESM.docx]

Journal of Autism and Developmental Disorders

Supplementary Material

Prediction of autism at 3 years from behavioural and developmental measures in high-risk infants: a longitudinal cross-domain classifier analysis

Bussu G. ^*^, Jones E.J.H., Charman T., Johnson M.H., Buitelaar, J.K. and the BASIS Team.

^*^ corresponding author: Giorgia Bussu, [g.bussu@donders.ru.nl](mailto:g.bussu@donders.ru.nl)

Donders Centre for Cognitive Neuroimaging, Radboudumc

Kapittelweg 29, 6525 EN Nijmegen

Supplementary Methods and Results

Missing Data

Data presented in the current paper were collected as part of a large longitudinal study, to which 247 infants participated in one of two phases of longitudinal assessments (104 in Phase 1 and 143 in Phase 2). Missing data was mainly due to non-attendance to visits. N=10 infants were excluded from this study because they did not receive an ADOS (Autism Diagnostic Observation Schedule) evaluation and/or a clinical outcome evaluation at 36 months. We investigated the pattern of missing data in the selected sample (N=237) at different time-points, testing differences on risk-group (high-risk siblings, HR; low-risk controls, LR), gender, age and clinical outcome at 36 months (LR; *HR-Typical*; *HR-Atypical*; *HR-ASD*) between infants with complete and missing data. Table S1 shows the number of infants with complete data among those who attended the visit. We found a total of 3.5% of data missing, and differences on risk-group (*p*<0.001, *t*=-8.35, *df*=233), gender (*p*=0.04, *t*=2.05, *df*=233), and clinical outcome (*p*<0.001, *t*=-6.62, *df*=233) were significant at 24 months, showing a pattern of data missing at random. We performed imputation through expectation maximization to handle missing data at a specific time-point. Analyses were performed on SPSS (<http://www.ibm.com/analytics/us/en/technology/spss>). Our aim was to obtain a longitudinally complete dataset for each infant between 8 and 36 months, thus infants who did not attend at least one of the visits were excluded from the study (N=5). Our final sample included 232 infants (161 [69.4%] HR and 71 [30.6%] LR). Infants excluded from the study (N=15) did not differ from the selected sample on risk group (*p*=0.45, *t*=0.76, *df*=245), gender (*p*=0.21, *t*=-1.25, *df*=245), and age at intake (*p*=0.53, *t*=-0.63, *df*=245), showing a pattern of data missing completely at random.

Atypical Clinical Outcome

Criteria for the atypical outcome were: ADOS above ASD (Autism Spectrum Disorder) threshold (N=31) or ADI-R (Autism Diagnostic Interview – Revised) above ASD threshold (N=6) or MSEL (Mullen Scales of Early Learning) more than 1.5 standard deviations below the average on visual reception (N=7), receptive language (N=13), expressive language (N=9), or early learning composite score (N=15). Among these, N=20 infants met only the ADOS criterion; N=11 met only the MSEL criterion; N=1 met only the ADI-R criterion; N=6 met ADOS and MSEL criteria; N=4 met ADOS and ADI-R criteria; and N=1 met all criteria.

Classifiers

To predict autism at pre-diagnostic ages, we performed a classifier analysis using scores from MSEL, VABS and AOSI as features. Twenty-two classifiers were tested, and features for each classifier are shown in Table S2.

Intra-individual Variability

Pearson correlation between measures at 8 and 14 months by clinical outcome groups were computed as a measure of intra-individual variability in developmental trajectories between the time-point used for classification. Analyses were performed on Matlab R2016b (MATLAB 9.1, The MathWorks Inc., Natick, MA, 2016). Results are shown in Table S3.

Developmental trajectories.

*Gross motor score.* The quadratic model was unidentifiable having only 3 time points available for measurements, thus we chose the linear model for data modeling. Furthermore, the model with interaction between outcome and age was not significantly better than the model without interaction effects (χ^2^(3)=1.3, p=0.72), showing no interaction effect between age and outcome for gross motor scores. We found a significant main effect of age (*F*(1,471.2)=15.5, *p*<0.001), and outcome (*F*(3, 227.2)=4.8, *p*<0.005) on Mullen gross motor scores. Post-hoc group comparisons showed LR having higher scores than *HR-Atypical* (p=0.018) and *HR-ASD* (p=0.007).

*Fine motor score.* Quadratic model fitting was significantly better than linear fitting (χ^2^(7)=42.7, p<0.001), and similarly the model with interaction between outcome and age was better than the model without interaction effects (χ^2^(9)=41.2, p<0.001). We found a significant main effect of age (*F*(1,227.6)=40.0, *p*<0.001), age^2^ (*F*(1,330.4)=4.00, *p*<0.05) and outcome (*F*(3, 304.7)=8.2, *p*<0.001) on Mullen fine motor scores. There was also a significant effect of gender covariate (*F*(1,234.7)=9.2, *p*<0.005) and an interaction effect of age^2^ with outcome (*F*(3,324.0)=4.0, *p*<0.005). Post-hoc group comparisons on simple main effects showed LR having higher scores than *HR-ASD* at all time-points (p<0.05 at 8 months, p<0.005 at 14 and 24 months, p<0.001 at 36 months), higher scores than *HR-Typical* at 24 months (p<0.05), and higher scores than *HR-Atypical* at 14 (p<0.05), 24 (p<0.005) and 36 months (p<0.001). Furthermore, *HR-Typical* had higher scores than *HR-Atypical* (trend level, p=0.07) and *HR-ASD* (p<0.05) at 36 months.

*Receptive language score.* Quadratic model fitting was significantly better than linear fitting (χ^2^(7)=23.5, p<0.005), and similarly the model with interaction between outcome and age was better than the model without interaction effects (χ^2^(9)=44.4, p<0.001). We found a significant main effect of age (*F*(1,229.1)=30.0, *p*<0.001) and outcome (*F*(3, 272)=15.1, *p*<0.001) on Mullen receptive language scores. There was also a significant effect of gender covariate (*F*(1,231.8)=9.4, *p*<0.005) and an interaction effect of age (*F*(3,228.7)=7.8, *p*<0.001) and age^2^ with outcome (*F*(3,588.4)=5.0, *p*<0.005). Post-hoc group comparisons on simple main effects showed LR having higher scores than *HR-Atypical* at 14 (p<0.05), 24 and 36 months (p<0.001), and higher scores than *HR-ASD* from 14 months onwards (p<0.001). Furthermore, *HR-Typical* had higher scores than *HR-Atypical* at 36 months (p<0.001), and higher scores than *HR-ASD* from 14 months onwards (p<0.05).

*Expressive language score.* Quadratic model fitting was significantly better than linear fitting (χ^2^(7)=40.0, p<0.001), and similarly the model with interaction between outcome and age was better than the model without interaction effects (χ^2^(9)=66.2, p<0.001). We did not find a significant main effect of age or age^2^, but a significant main effect of outcome (*F*(3, 244.8)=9.6, *p*<0.001) and gender covariate (*F*(1,227.4)=4.1, *p*<0.05). Furthermore, we found an interaction effect of age with outcome (*F*(3,227.1)=9.1, *p*<0.001). Post-hoc group comparisons on simple main effects showed LR having higher scores than *HR-Atypical* at 24 (p<0.005) and 36 months (p<0.001), and higher scores than *HR-ASD* at 14 months (p<0.05), 24 and 36 months (p<0.001). Furthermore, *HR-Typical* had higher scores than *HR-Atypical* and *HR-ASD* at 36 months (p<0.005).

*Visual reception score.* Quadratic model fitting was significantly better than linear fitting (χ^2^(7)=20.3, p=0.005), and similarly the model with interaction between outcome and age was better than the model without interaction effects (χ^2^(9)=19.0, p=0.03). We found a significant main effect of age^2^ (*F*(1,235.0)=11.9, *p*<0.001) and outcome (*F*(3, 225.0)=12.7, *p*<0.001). There was also a significant effect of gender covariate (*F*(1,226.7)=10.2, *p*<0.005) and an interaction effect of age (*F*(3,220.8)=3.1, *p*=0.03) and age^2^ with outcome (*F*(3,218.9)=2.7, *p*=0.05). Post-hoc group comparisons on simple main effects showed LR having higher scores than *HR-Typical* at 14 months (p<0.05), higher scores than *HR-Atypical* from 14 months onwards (p<0.001), and higher scores than *HR-ASD* at 14 and 24 months (p<0.001) and 36 months (p<0.05). Furthermore, *HR-Typical* at 36 months had higher scores than *HR-Atypical* (p<0.005) and *HR-ASD* (p<0.05).

*Communication score.* Quadratic model fitting was significantly better than linear fitting (χ^2^(7)=43.3, p<0.001), and similarly the model with the interaction between outcome and age^2^ was better than the model without interaction effects (χ^2^(3)=10.2, p=0.017). We found a significant main effect of age (*F*(1,222.7)=38.4, *p*<0.001), age^2^ (*F*(1,274.6)=13.8, *p*<0.001) and outcome (*F*(3, 242.4)=22.4, *p*<0.001) on Vineland communication scores. There was also a significant effect of gender covariate (*F*(1,227.2)=13.3, *p*<0.001) and an interaction effect of age^2^ with outcome (*F*(3,195.2)=3.5, *p*=0.017). Post-hoc group comparisons on simple main effects showed LR having higher scores than *HR-Atypical* and *HR-ASD* at all time-points (p<0.001, except for *HR-Atypical* at 24 months with p<0.005), and higher scores than *HR-Typical* at 36 months (p<0.05); and *HR-Typical* having higher scores than *HR-Atypical* at 8 (p<0.05) and 36 months (p<0.005), and higher scores than *HR-ASD* at all time-points (*p*<0.001 at 8 months, p<0.05 at 14 and 24 months, and p<0.005 at 36 months).

*Daily living score.* Quadratic model fitting was not significantly better than linear fitting (χ^2^(7)=9.7, p=0.21), thus the linear model was selected for model fitting. Furthermore, the model with interaction between outcome and age was better than the model without interaction effects (χ^2^(3)=9.6, p=0.02). We found a significant main effect of age (*F*(1,227.4)=20.5, *p*<0.001), and outcome (*F*(3, 227)=15.9, *p*<0.001) on Vineland daily living scores. There was also a significant effect of gender covariate (*F*(1,226.9)=9.0, *p*<0.005) and an interaction effect of age with outcome (*F*(3,225.2)=3.2, *p*=0.02). Post-hoc group comparisons on simple main effects showed LR having higher scores than *HR-ASD* at all time-points (p<0.001), and higher scores than *HR-Atypical* at 24 and 36 months (p<0.001); and *HR-Typical* having higher scores than *HR-Atypical* at 24 and 36 months (p<0.05), and higher scores than *HR-ASD* at 14 (*p*<0.05), 24 and 36 months (p<0.001).

*Social score.* Quadratic model fitting was not significantly better than linear fitting (χ^2^(7)=11.5, p=0.12), thus the linear model was selected for model fitting. Furthermore, the model with interaction between outcome and age was better than the model without interaction effects (χ^2^(3)=273.5, p<0.001). We found a significant main effect of age (*F*(1,226.9)=11.9, *p*<0.001), and outcome (*F*(3, 227.2)=23.2, *p*<0.001) on Vineland social scores. There was also a significant effect of gender covariate (*F*(1,226.3)=9.7, *p*<0.005) and an interaction effect of age with outcome (*F*(3,225.5)=14.7, *p*<0.001). Post-hoc group comparisons on simple main effects showed LR having higher scores than *HR-Typical* at 24 and 36 months (p<0.05), *HR-Atypical* at 14 (p<0.05), 24 and 36 months (p<0.001), and *HR-ASD* from 14 months onwards (p<0.001); *HR-Atypical* having higher scores than *HR-ASD* at 24 (*p*<0.05) and 36 months (p<0.001); and *HR-Typical* having higher scores than *HR-Atypical* at 36 months (p<0.05), and *HR-ASD* at 24 and 36 months (p<0.001).

*Motor score.* Quadratic model fitting was significantly better than linear fitting (χ^2^(7)=183.4, p<0.001), and similarly the model with interaction between outcome and age was better than the model without interaction effects (χ^2^(9)=121.2, p<0.001). We found a significant main effect of age (*F*(1,223.7)=25.6, *p*<0.001), age^2^ (*F*(1,242.9)=187.1, *p*<0.001), but only marginally significant main effect of outcome (*F*(3, 225.5)=2.6, *p*=0.053) on Vineland motor scores. There was also a significant effect of gender covariate (*F*(1,224.4)=6.1, *p*=0.014) and an interaction effect of age^2^ with outcome (*F*(3,223.2)=10.6, *p*<0.001). Post-hoc group comparisons on simple main effects showed LR having higher scores than *HR-Atypical* at 8 (p<0.001),14 (p<0.05), and 36 months (p<0.001), and higher scores than *HR-ASD* at 36 months (p<0.001); and *HR-Typical* having higher scores than *HR-Atypical* (p<0.05) and *HR-ASD* at 36 months (p<0.005).

Classifier Analysis

*Prediction of HR-ASD vs. HR-Atypical and HR-Typical.* Table S4 shows performance measured by Area Under the Curve (AUC) of different classifiers at predicting *HR-ASD* vs. other high-risk siblings. Classification before 14 months was not significantly different from chance level, thus we did not perform comparative analyses on the performance of those classifiers. Using measures at 14 months, prediction was significantly different from random, thus we performed a nonparametric Friedman test on classifier performance (AUC) at 14 months. We found a significant difference in classifier performance, χ^2^(21)=117, *p*<0.001; yet post-hoc comparisons through paired Wilcoxon test missed significance after Bonferroni correction for multiple comparison (α_Bonferroni_=0.0024; see Table S5). The classifier with highest AUC was built on daily living scores at 14 month, yet paired Wilcoxon tests between this classifier and the ones with highest AUC at 8 months (motor scores) and 8 months plus the change factor between 8 and 14 months (motor + social + daily living scores) missed significance (respectively *z*=-1.9, *p*=0.06; *z*=-1.7, *p*=0.09). We also tested significant changes on performance of the same classifier over time by means of a nonparametric Friedman test, and Bonferroni corrected post-hoc paired Wilcoxon tests (3 pairs, α_Bonferroni_=0.017). Results are shown in Table S6.

*Prediction of HR-ASD + HR-Atypical vs. HR-Typical.* Table S7 shows performance measured by AUC of different classifiers at predicting *HR-ASD* and *HR-Atypical* vs. typically developing siblings at 8 months, 8 months adding the change factor between 8 and 14 months, and 14 months. We found a significant difference in classifier performance at 8 months, χ^2^(21)=137, *p*<0.001; 8 months with the addition of the change factor between 8 and 14 months, χ^2^(21)=131, *p*<0.001; and 14 months, χ^2^(21)=105, p<0.001. Results from post-hoc paired Wilcoxon tests are shown in Table S8. The classifier with highest AUC was built on the integration of Vineland and AOSI scores at 14 month, yet paired Wilcoxon tests between this classifier and the ones with highest AUC at 8 months and 8 months plus the change factor between 8 and 14 months (motor + communication scores) missed significance (respectively *z*=-0.7, *p*=0.5; *z*=-0.8, *p*=0.4). We also tested significant changes on performance of the same classifier over time by means of a nonparametric Friedman test, and Bonferroni corrected post-hoc paired Wilcoxon tests (3 pairs, α_Bonferroni_=0.017). Results are shown in Table S9.

We also compared performance of the classifiers with highest AUC at each time-point between the two different classification problems (prediction of *HR-ASD* and *HR-ASD + HR-Atypical*) by means of paired Wilcoxon tests. Differences missed significance. We compared at 8 months the motor classifier for ASD and the motor + communication classifier for atypical classification (*z*=-1.1, *p*=0.3); at 8 months plus change factor, the motor + social + daily living classifier for ASD classification vs. the motor + communication classifier for atypical classification (*z*=-0.8, *p*=0.4); at 14 months the daily living classifier for ASD classification vs. the VABS + AOSI classifier for atypical classification (*z*=-0.3, *p*=0.8).

| Visit | Attendance (*n/n_total*) | Complete subjects (*n/n_attendance*) |
| --- | --- | --- |
| 8 months | 237/237 | 231/237 |
| 14 months | 234/237 | 223/234 |
| 24 months | 235/237 | 158/235 |
| 36 months | 237/237 | 230/237 |

**Table S1. Missing data.** This table shows in the second column the number of infants attending each visit (*n/n_total*), where *n_total*=237 is the total number of infants after excluding infants who did not receive a clinical evaluation and/or an ADOS classification at 36 months. In the third column, the number of infants with all complete scores is shown over the number of participants for each visit (*n_attendance*).

| Classifier | Features |
| --- | --- |
| msel | GM + FM + RL + EL + VR |
| vabs | Comm + DL + Mot + Soc |
| aosi | AOSI total score |
| msel + vabs | GM + FM + RL + EL + VR + Comm + DL + Mot + Soc |
| msel + aosi | GM + FM + RL + EL + VR + AOSI total score |
| vabs + aosi | Comm + DL + Mot + Soc + AOSI total score |
| all instruments | GM + FM + RL + EL + VR + Comm + DL + Mot + Soc + AOSI total score |
| motor | GM + FM + Mot |
| communication | RL + EL + Comm |
| daily living | DL |
| social | Soc |
| motor + communication | GM + FM + Mot + RL + EL + Comm |
| motor + social | GM + FM + Mot + Soc |
| communication + social | RL + EL + Comm + Soc |
| motor + daily living | GM + FM + Mot + DL |
| communication + daily living | RL + EL + Comm + DL |
| social + daily living | Soc + DL |
| motor + communication + daily living | GM + FM + Mot + RL + EL + Comm + DL |
| motor + social + daily living | GM + FM + Mot + Soc + DL |
| communication + social + daily living | RL + EL + Comm + Soc + DL |
| motor + communication + social | GM + FM + Mot + RL + EL + Comm + Soc |
| all domains | GM + FM + Mot + RL + EL + Comm + Soc + DL |

**Table S2. Classifiers.** This table describes the feature composition of classifiers used to predict ASD and atypical outcome at 36 months. Abbreviations: ASD= autism spectrum disorder; MSEL= Mullen Scales of Early Learning; GM= gross motor abilities (MSEL); FM= fine motor abilities (MSEL); VR= visual reception (MSEL); RL= receptive language (MSEL); EL= expressive language (MSEL); VABS = Vineland Adaptive Behavior Scales; Comm = communication skills (VABS); DL = daily living skills (VABS); Soc = social skills (VABS); Mot = motor skills (VABS); AOSI= Autism Observation Scale for Infants.

| Measure | HR-ASD  (n=32) | HR-Atypical  (n=43) | HR-Typical  (n=86) |
| --- | --- | --- | --- |
| GM | 0.67 | 0.23 | 0.52 |
| VR | 0.40 | 0.44 | 0.26 |
| FM | 0.11 | 0.50 | 0.34 |
| RL | 0.40 | -0.02 | 0.11 |
| EL | 0.43 | 0.34 | 0.17 |
| Comm | 0.18 | 0.37 | 0.40 |
| DL | 0.40 | 0.38 | 0.24 |
| Soc | 0.15 | 0.59 | 0.43 |
| Mot | 0.74 | 0.61 | 0.52 |
| AOSI total score | 0.15 | 0.35 | 0.29 |

**Table S3. Intra-individual variability between 8 and 14 months.** This table reports the Pearson correlation coefficient between single measures at 8 and 14 months by clinical outcome groups (LR, HR-Typical, HR-Atypical, HR-ASD). The number of infants in each group is also reported (*n*). Abbreviations: HR= high-risk siblings; LR= low-risk controls; ASD= autism spectrum disorder; MSEL= Mullen Scales of Early Learning; GM= gross motor abilities (MSEL); FM= fine motor abilities (MSEL); VR= visual reception (MSEL); RL= receptive language (MSEL); EL= expressive language (MSEL); VABS = Vineland Adaptive Behavior Scales; Comm = communication skills (VABS); DL = daily living skills (VABS); Soc = social skills (VABS); Mot = motor skills (VABS); AOSI= Autism Observation Scale for Infants.

| Classifier | 8 months | | 8 months + change factor | | 14 months | |
| --- | --- | --- | --- | --- | --- | --- |
|  | *p* | AUC  (%) | *p* | AUC  (%) | *p* | AUC  (%) |
| msel | 0.21 | 61.3  (42.5, 78.9) | 0.13 | 64.8  (47.3, 81.3) | 0.10 | 64.2  (43.1, 83.0) |
| vabs | 0.31 | 57.4  (38.0, 75.6) | 0.23 | 59.4  (39.8, 77.9) | 0.047^*^ | 70.1  (52.7, 84.9) |
| aosi | 0.25 | 57.4  (37.8, 18.2) | 0.22 | 58.5  (38.6, 77.9) | 0.13 | 62.0  (45.8, 77.3) |
| msel + vabs | 0.32 | 55.7  (35.7, 74.6) | 0.17 | 61.8  (41.6, 80.6) | 0.07 | 67.9  (50.0, 84.3) |
| msel + aosi | 0.54 | 49.5  (30.1, 68.7) | 0.32 | 56.5  (37.4, 75.6) | 0.34 | 56.6  (38.0, 74.3) |
| vabs + aosi | 0.30 | 56.0  (35.4, 75.8) | 0.24 | 58.3  (37.5, 77.4) | 0.04^*^ | 70.6  (53.1, 85.6) |
| all instruments | 0.28 | 56.0  (36.7, 74.7) | 0.25 | 57.5  (38.1, 75.7) | 0.32 | 58.1  (39.7, 76.0) |
| motor | 0.11 | 65.1  (46.6, 82.5) | 0.12 | 65.8  (48.1, 82.1) | 0.30 | 56.5  (37.2, 74.3) |
| communication | 0.24 | 58.3  (39.0, 76.7) | 0.17 | 61.7  (42.2, 79.4) | 0.03^*^ | 69.0  (50.5, 85.7) |
| daily living | 0.21 | 60.7  (41.6, 78.7) | 0.14 | 63.4  (44.0, 81.0) | 0.03^*^ | 71.3  (55.6, 85.1) |
| social | 0.58 | 47.6  (28.3, 66.4) | 0.64 | 45.9  (26.0, 66.1) | 0.14 | 62.6  (45.4, 78.9) |
| motor + communication | 0.17 | 61.5  (42.5, 79.1) | 0.16 | 62.5  (42.7, 80.7) | 0.27 | 59.2  (39.7, 77.9) |
| motor + social | 0.19 | 61.0  (42.4, 78.3) | 0.10 | 64.7  (46.8, 80.9) | 0.12 | 64.4  (46.9, 80.3) |
| communication + social | 0.27 | 57.7  (38.8, 75.3) | 0.22 | 59.3  (39.6, 77.8) | 0.047^*^ | 68.3  (51.0, 84.1) |
| motor + daily living | 0.14 | 64.4  (44.5, 81.9) | 0.18 | 63.6  (43.7, 81.2) | 0.04^*^ | 70.0  (53.3, 85.1) |
| communication + daily living | 0.21 | 60.2  (38.9, 80.1) | 0.18 | 61.6  (41.4, 80.0) | 0.02^*^ | 70.8  (53.4, 86.1) |
| social + daily living | 0.26 | 59.6  (39.5, 78.6) | 0.21 | 60.5  (40.3, 79.3) | 0.07 | 68.6  (51.7, 83.4) |
| motor + communication + daily living | 0.17 | 61.2  (40.8, 79.8) | 0.22 | 60.4  (41.2, 77.8) | 0.41 | 52.3  (31.7, 72.9) |
| motor + social + daily living | 0.17 | 62.5  (42.3, 80.5) | 0.10 | 65.9  (47.0, 83.3) | 0.06 | 68.9  (51.7, 83.9) |
| communication + social + daily living | 0.25 | 59.2  (39.2, 78.3) | 0.20 | 61.7  (42.0, 80.0) | 0.09 | 67.1  (49.5, 82.9) |
| motor + communication + social | 0.24 | 59.6  (41.2, 76.9) | 0.16 | 61.6  (43.2, 78.5) | 0.37 | 54.3  (33.5, 74.2) |
| all domains | 0.38 | 54.6  (34.6, 73.8) | 0.15 | 62.0  (41.9, 80.4) | 0.46 | 51.5  (30.8, 72.4) |

**Table S4. Classifier performances for prediction of ASD outcome at 36 months.** Predictive performance as measured by the AUC of the classifiers using as features developmental, behavioural and symptoms measures for classifying HR siblings who later develop ASD from their typically developing and atypical non-ASD peers (*HR-ASD vs HR-Typical + HR-Atypical*). The significance of classification AUC was determined by permutation test, the resulting p-values are reported. Prediction was considered different from chance level if *p*<0.05 (marked as ^*^). 95% confidence interval is reported in parentheses. Measures are reported as *mean (lower level CI, upper level CI)*. Abbreviations: AUC = area under the curve; MSEL = Mullen Scales of Early Learning (5 scores); VABS = Vineland Adaptive Behavior Scales (4 scores); AOSI = Autism Observation Scale for Infants, in this study we considered the total score.

| Paired classifiers (daily living vs.) | *z* | *p* |
| --- | --- | --- |
| msel | -2.19 | 0.028 |
| vabs | -0.92 | 0.359 |
| aosi | -2.50 | 0.013 |
| msel + vabs | -1.17 | 0.241 |
| msel + aosi | -2.40 | 0.017 |
| vabs + aosi | -0.30 | 0.767 |
| all instruments | -2.55 | 0.011 |
| motor | -2.80 | 0.005 |
| communication | -0.97 | 0.333 |
| social | -2.80 | 0.005 |
| motor + communication | -2.09 | 0.037 |
| motor + social | -2.40 | 0.017 |
| communication + social | -1.17 | 0.241 |
| motor + daily living | -1.07 | 0.285 |
| communication + daily living | -0.46 | 0.646 |
| social + daily living | -2.50 | 0.013 |
| motor + communication + daily living | -2.80 | 0.005 |
| motor + social + daily living | -2.04 | 0.041 |
| communication + social + daily living | -1.07 | 0.285 |
| motor + communication + social | -2.80 | 0.005 |
| all domains | -2.80 | 0.005 |

**Table S5. Paired Wilcoxon tests on classifier performance for ASD prediction at 14 months.** This table shows results from paired Wilcoxon tests (*z-score* and two-tailed *p-value*) on predictive performance, measured by Area Under the Curve (AUC), of the classifier of interest (daily living score at 14 months) and the other classifiers built on measures at the same time-point (14 months). Paired tests were performed as post-hoc analysis for significant Friedman tests on predictive performance of classifiers at the same time-point. Bonferroni correction was used to correct for multiple comparisons (21 pairs), and results were significant for *p*<α_Bonferroni_, with α_Bonferroni_==0.0024. Abbreviations: MSEL= Mullen Scales of Early Learning; VABS = Vineland Adaptive Behavior Scales; AOSI= Autism Observation Scale for Infants.

| Classifier | Friedman Test | | | Wilcoxon Tests | | | | | |  |
| --- | --- | --- | --- | --- | --- | --- | --- | --- | --- | --- |
|  |  |  |  | 8m + slope vs. 8m | | 14m vs. 8m | | 14m vs. 8m + slope | | |
|  | *χ^2^* | *df* | *p* | *z* | *p* | *z* | *p* | *z* | *p* |  |
| msel | 1.90 | 2 | 0.39 | . | . | . | . | . | . |  |
| vabs | 10.40 | 2 | 0.006 | -1.68 | 0.09 | -2.60 | 0.009^*^ | -2.70 | 0.007^*^ |  |
| aosi | 3.80 | 2 | 0.15 | . | . | . | . | . | . |  |
| msel + vabs | 9.80 | 2 | 0.007 | -2.80 | 0.005^*^ | -2.50 | 0.013^*^ | -1.89 | 0.059 |  |
| msel + aosi | 1.80 | 2 | 0.41 | . | . | . | . | . | . |  |
| vabs + aosi | 13.40 | 2 | 0.001 | -2.09 | 0.036 | -2.70 | 0.007^*^ | -2.80 | 0.005^*^ |  |
| all instruments | 0.36 | 2 | 0.84 | . | . | . | . | . | . |  |
| motor | 12.67 | 2 | 0.002 | -0.59 | 0.55 | -2.70 | 0.007^*^ | -2.81 | 0.005^*^ |  |
| communication | 14.60 | 2 | 0.001 | -2.70 | 0.007^*^ | -2.80 | 0.005^*^ | -2.50 | 0.013^*^ |  |
| daily living | 7.80 | 2 | 0.02 | -2.40 | 0.017^*^ | -2.50 | 0.013^*^ | -2.09 | 0.037 |  |
| social | 13.40 | 2 | 0.001 | -0.76 | 0.45 | -2.70 | 0.007^*^ | -2.80 | 0.005^*^ |  |
| motor + communication | 0.60 | 2 | 0.74 | . | . | . | . | . | . |  |
| motor + social | 3.80 | 2 | 0.15 | . | . | . | . | . | . |  |
| communication + social | 12.60 | 2 | 0.002 | -1.07 | 0.28 | -2.70 | 0.007^*^ | -2.80 | 0.005^*^ |  |
| motor + daily living | 4.97 | 2 | 0.08 | . | . | . | . | . | . |  |
| communication + daily living | 14.60 | 2 | 0.001 | -1.48 | 0.14 | -2.80 | 0.005^*^ | -2.70 | 0.007^*^ |  |
| social + daily living | 5.60 | 2 | 0.06 | . | . | . | . | . | . |  |
| motor + communication + daily living | 8.60 | 2 | 0.014 | -0.66 | 0.51 | -2.50 | 0.013^*^ | -1.87 | 0.059 |  |
| motor + social + daily living | 7.40 | 2 | 0.025 | -2.81 | 0.005^*^ | -1.99 | 0.047 | -1.38 | 0.17 |  |
| communication + social + daily living | 9.39 | 2 | 0.009 | -2.40 | 0.017^*^ | -1.79 | 0.07 | -1.48 | 0.14 |  |
| motor + communication + social | 3.80 | 2 | 0.15 | . | . | . | . | . | . |  |
| all domains | 7.40 | 2 | 0.03 | -1.78 | 0.07 | -0.76 | 0.45 | -2.60 | 0.009^*^ |  |

**Table S6. ASD classifier performance over time.** This table shows results from Friedman test on classifier performance over time (8 months, 8 months + change factor between 8 and 14 months, 14 months) measured by Area Under the Curve (AUC). Post-hoc paired Wilcoxon tests were performed when the effect of time was significant (*p*<0.05 from Friedman test). Results are reported. Bonferroni correction was used to correct for multiple comparisons (3 pairs), and results were significant (^*^) for *p*<α_Bonferroni_, with α_Bonferroni_==0.017. Abbreviations: MSEL= Mullen Scales of Early Learning; VABS = Vineland Adaptive Behavior Scales; AOSI= Autism Observation Scale for Infants.

| Classifier | 8 months | | 8 months + change factor | | 14 months | |
| --- | --- | --- | --- | --- | --- | --- |
|  | *p* | AUC  (%) | *p* | AUC  (%) | *p* | AUC  (%) |
| msel | 0.02^*^ | 66.5  (52.6, 79.8) | 0.01^*^ | 67.7  (53.8, 80.9) | 0.05 | 64.6  (50.3, 77.9) |
| vabs | 0.047^*^ | 63.9  (49.7, 77.3) | 0.04^*^ | 65.1  (51.2, 78.0) | 0.05 | 65.1  (50.7, 78.4) |
| aosi | 0.24 | 56.0  (41.8, 69.4) | 0.09 | 62.3  (47.5, 75.7) | 0.01^*^ | 69.8  (56.5, 82.1) |
| msel + vabs | 0.02^*^ | 66.6  (53.0, 79.1) | 0.01^*^ | 67.8  (53.9, 80.6) | 0.03^*^ | 66.9  (52.7, 79.8) |
| msel + aosi | 0.02^*^ | 67.1  (53.2, 80.2) | 0.01^*^ | 68.7  (54.9, 81.5) | 0.03^*^ | 67.6  (53.8, 80.8) |
| vabs + aosi | 0.04^*^ | 64.8  (50.6, 78.0) | 0.02^*^ | 66.8  (52.8, 79.6) | 0.01^*^ | 70.8  (57.1, 83.2) |
| all instruments | 0.01^*^ | 67.6  (53.6, 80.1) | 0.01^*^ | 68.8  (55.0, 81.5) | 0.02^*^ | 69.1  (55.1, 82.0) |
| motor | 0.02^*^ | 65.9  (51.2, 68.6) | 0.02^*^ | 66.8  (52.6, 79.5) | 0.07 | 62.6  (48.3, 76.3) |
| communication | 0.03^*^ | 65.9  (52.2, 78.9) | 0.01^*^ | 67.2  (53.1, 79.8) | 0.02^*^ | 68.1  (53.9, 80.9) |
| daily living | 0.24 | 56.2  (42.0, 70.2) | 0.19 | 56.7  (41.8, 71.0) | 0.07 | 64.2  (50.3, 77.6) |
| social | 0.42 | 51.6  (37.0, 66.0) | 0.29 | 54.5  (39.7, 68.6) | 0.17 | 58.5  (43.8, 72.6) |
| motor + communication | 0.01^*^ | 69.2  (55.6, 81.8) | 0.01^*^ | 69.4  (55.6, 82.1) | 0.02^*^ | 67.5  (53.4, 80.2) |
| motor + social | 0.03^*^ | 64.9  (50.9, 78.1) | 0.02^*^ | 66.2  (52.0, 79.0) | 0.07 | 63.6  (48.8, 76.3) |
| communication + social | 0.03^*^ | 65.1  (50.6, 78.4) | 0.02^*^ | 66.8  (53.1, 79.4) | 0.02^*^ | 67.8  (54.2, 80.4) |
| motor + daily living | 0.03^*^ | 64.3  (50.2, 77.4) | 0.03^*^ | 64.7  (50.6, 77.7) | 0.04^*^ | 64.8  (50.7, 77.8) |
| communication + daily living | 0.04^*^ | 64.5  (50.5, 77.7) | 0.03^*^ | 66.0  (51.6, 78.7) | 0.02^*^ | 68.5  (54.4, 81.5) |
| social + daily living | 0.29 | 54.9  (40.1, 68.9) | 0.24 | 55.6  (40.8, 69.9) | 0.08 | 63.0  (48.6, 76.4) |
| motor + communication + daily living | 0.01^*^ | 68.2  (54.6, 81.0) | 0.01^*^ | 68.7  (54.9, 81.3) | 0.02^*^ | 68.1  (53.9, 81.0) |
| motor + social + daily living | 0.047^*^ | 63.3  (49.1, 76.5) | 0.03^*^ | 64.6  (50.4, 77.7) | 0.05 | 63.9  (49.8, 77.5) |
| communication + social + daily living | 0.06 | 63.6  (49.2, 76.7) | 0.04^*^ | 65.3  (51.7, 78.3) | 0.03^*^ | 67.4  (53.6, 80.3) |
| motor + communication + social | 0.01^*^ | 68.5  (55.2, 81.3) | 0.01^*^ | 69.3  (55.5, 82.0) | 0.02^*^ | 67.2  (52.5, 80.3) |
| all domains | 0.01^*^ | 67.7  (53.9, 80.6) | 0.01^*^ | 68.3  (54.5, 80.8) | 0.02^*^ | 67.7  (53.7, 80.6) |

**Table S7. Classifier performances for prediction of ASD plus atypical outcome at 36 months.** Predictive performance as measured by the AUC of the classifiers using as features developmental, behavioural and symptoms measures for classifying HR siblings who later develop ASD or with other atypical development from their typically developing (*HR-ASD + HR-Atypical vs. HR-Typical*). The significance of classification AUC was determined by permutation test, the resulting p-values are reported. Prediction was considered different from chance level if *p*<0.05 (marked as ^*^). 95% confidence interval is reported in parentheses. Measures are reported as *mean (lower level CI, upper level CI)*. Abbreviations: AUC = area under the curve; MSEL = Mullen Scales of Early Learning (5 scores); VABS = Vineland Adaptive Behavior Scales (4 scores); AOSI = Autism Observation Scale for Infants, in this study we considered the total score.

| Paired classifiers | motor + communication  (8 months) | | | motor + communication  (8 months+change factor) | | VABS + AOSI  (14 months) | |
| --- | --- | --- | --- | --- | --- | --- | --- |
|  | *z* | | *p* | *z* | *p* | *z* | *p* |
| msel | | -1.99 | 0.047 | -1.68 | 0.09 | -2.80 | 0.005 |
| vabs | | -2.50 | 0.013 | -2.40 | 0.017 | -2.80 | 0.005 |
| aosi | | -2.80 | 0.005 | -2.50 | 0.013 | -0.46 | 0.65 |
| msel + vabs | | -2.29 | 0.022 | -2.35 | 0.019 | -2.70 | 0.007 |
| msel + aosi | | -1.68 | 0.093 | -0.56 | 0.58 | -1.68 | 0.09 |
| vabs + aosi | | -2.29 | 0.022 | -1.68 | 0.09 | . | . |
| all instruments | | -1.78 | 0.074 | -0.97 | 0.33 | -0.97 | 0.33 |
| motor | | -2.70 | 0.007 | -2.70 | 0.007 | -2.80 | 0.005 |
| communication | | -2.09 | 0.037 | -2.29 | 0.022 | -1.89 | 0.059 |
| social | | -2.80 | 0.005 | -2.80 | 0.005 | -2.80 | 0.005 |
| daily living | | -2.80 | 0.005 | -2.80 | 0.005 | -2.60 | 0.009 |
| motor + communication | | . | . | . | . | -2.40 | 0.017 |
| motor + social | | -2.80 | 0.005 | -2.70 | 0.007 | -2.81 | 0.005 |
| communication + social | | -2.60 | 0.009 | -2.70 | 0.007 | -2.29 | 0.005 |
| motor + daily living | | -2.70 | 0.007 | -2.81 | 0.005 | -2.70 | 0.007 |
| communication + daily living | | -2.19 | 0.028 | -2.29 | 0.022 | -1.60 | 0.11 |
| social + daily living | | -2.80 | 0.005 | -2.80 | 0.005 | -2.80 | 0.005 |
| motor + communication + daily living | | -1.48 | 0.14 | -1.84 | 0.06 | -1.99 | 0.047 |
| motor + social + daily living | | -2.80 | 0.005 | -2.80 | 0.005 | -2.80 | 0.005 |
| communication + social + daily living | | -2.35 | 0.019 | -2.40 | 0.017 | -2.70 | 0.007 |
| motor + communication + social | | -2.20 | 0.028 | -1.07 | 0.28 | -2.50 | 0.013 |
| all domains | | -1.99 | 0.047 | -2.30 | 0.022 | -2.50 | 0.013 |

**Table S8. Paired Wilcoxon tests on classifier performance for ASD and atypical outcome prediction.** This table shows results from paired Wilcoxon tests (*z-score* and two-tailed *p-value*) on predictive performance, measured by Area Under the Curve (AUC), of the classifier of interest (motor and communication scores at 8 months and 8 months plus the change factor between 8 and 14 months; VABS and AOSI scores at 14 months) and the other classifiers built on measures at the same time-point. Paired tests were performed as post-hoc analysis for significant Friedman tests on predictive performance of classifiers at the same time-point. Bonferroni correction was used to correct for multiple comparisons (21 pairs), and results were significant for *p*<α_Bonferroni_, with α_Bonferroni_==0.0024. MSEL= Mullen Scales of Early Learning; VABS = Vineland Adaptive Behavior Scales; AOSI= Autism Observation Scale for Infants.

| Classifier | Friedman Test | | | Wilcoxon Tests | | | | | |  |
| --- | --- | --- | --- | --- | --- | --- | --- | --- | --- | --- |
|  |  |  |  | 8m + slope vs. 8m | | 14m vs. 8m | | 14m vs. 8m + slope | | |
|  | *χ^2^* | *df* | *p* | *z* | *p* | *z* | *p* | *z* | *p* |  |
| msel | 3.2 | 2 | 0.2 | . | . | . | . | . | . |  |
| vabs | 2.0 | 2 | 0.4 | . | . | . | . | . | . |  |
| aosi | 12.8 | 2 | 0.002 | -2.7 | 0.007^*^ | -2.6 | 0.009^*^ | -2.5 | 0.013^*^ |  |
| msel + vabs | 1.8 | 2 | 0.4 | . | . | . | . | . | . |  |
| msel + aosi | 3.8 | 2 | 0.2 | . | . | . | . | . | . |  |
| vabs + aosi | 7.8 | 2 | 0.02 | -2.6 | 0.009^*^ | -1.9 | 0.06 | -1.5 | 0.1 |  |
| all instruments | 3.8 | 2 | 0.2 | . | . | . | . | . | . |  |
| motor | 7.2 | 2 | 0.03 | -2.1 | 0.04 | -1.9 | 0.06 | -2.0 | 0.05 |  |
| communication | 2.4 | 2 | 0.3 | . | . | . | . | . | . |  |
| daily living | 8.1 | 2 | 0.02 | -1.6 | 0.1 | -2,4 | 0.017^*^ | -2.4 | 0.017^*^ |  |
| social | 3.2 | 2 | 0.2 | . | . | . | . | . | . |  |
| motor + communication | 0.8 | 2 | 0.7 | . | . | . | . | . | . |  |
| motor + social | 2.4 | 2 | 0.3 | . | . | . | . | . | . |  |
| communication + social | 4.1 | 2 | 0.1 | . | . | . | . | . | . |  |
| motor + daily living | 0.8 | 2 | 0.7 | . | . | . | . | . | . |  |
| communication + daily living | 3.2 | 2 | 0.2 | . | . | . | . | . | . |  |
| social + daily living | 9.6 | 2 | 0.008 | -1.1 | 0.3 | -2.3 | 0.02 | -2.8 | 0.005^*^ |  |
| motor + communication + daily living | 1.4 | 2 | 0.5 | . | . | . | . | . | . |  |
| motor + social + daily living | 2.6 | 2 | 0.3 | . | . | . | . | . | . |  |
| communication + social + daily living | 4.2 | 2 | 0.1 | . | . | . | . | . | . |  |
| motor + communication + social | 2.6 | 2 | 0.3 | . | . | . | . | . | . |  |
| all domains | 1.4 | 2 | 0.5 | . | . | . | . | . | . |  |

**Table S9. ASD + atypical classifier performance over time.** This table shows results from Friedman test on classifier performance over time (8 months, 8 months + change factor between 8 and 14 months, 14 months) measured by Area Under the Curve (AUC). Post-hoc paired Wilcoxon tests were performed when the effect of time was significant (*p*<0.05 from Friedman test). Results are reported. Bonferroni correction was used to correct for multiple comparisons (3 pairs), and results were significant (^*^) for *p*<α_Bonferroni_, with α_Bonferroni_==0.017. Abbreviations: MSEL= Mullen Scales of Early Learning; VABS = Vineland Adaptive Behavior Scales; AOSI= Autism Observation Scale for Infants.
